# Negative Selection by an Endogenous Retrovirus Promotes a Higher-Avidity CD4^+^ T Cell Response to Retroviral Infection

**DOI:** 10.1371/journal.ppat.1002709

**Published:** 2012-05-10

**Authors:** George R. Young, Mickaël J.-Y. Ploquin, Urszula Eksmond, Munisch Wadwa, Jonathan P. Stoye, George Kassiotis

**Affiliations:** 1 Division of Immunoregulation, MRC National Institute for Medical Research, London, United Kingdom; 2 Division of Virology, MRC National Institute for Medical Research, London, United Kingdom; University of Pennsylvania School of Medicine, United States of America

## Abstract

Effective T cell responses can decisively influence the outcome of retroviral infection. However, what constitutes protective T cell responses or determines the ability of the host to mount such responses is incompletely understood. Here we studied the requirements for development and induction of CD4^+^ T cells that were essential for immunity to Friend virus (FV) infection of mice, according to their TCR avidity for an FV-derived epitope. We showed that a self peptide, encoded by an endogenous retrovirus, negatively selected a significant fraction of polyclonal FV-specific CD4^+^ T cells and diminished the response to FV infection. Surprisingly, however, CD4^+^ T cell-mediated antiviral activity was fully preserved. Detailed repertoire analysis revealed that clones with low avidity for FV-derived peptides were more cross-reactive with self peptides and were consequently preferentially deleted. Negative selection of low-avidity FV-reactive CD4^+^ T cells was responsible for the dominance of high-avidity clones in the response to FV infection, suggesting that protection against the primary infecting virus was mediated exclusively by high-avidity CD4^+^ T cells. Thus, although negative selection reduced the size and cross-reactivity of the available FV-reactive naïve CD4^+^ T cell repertoire, it increased the overall avidity of the repertoire that responded to infection. These findings demonstrate that self proteins expressed by replication-defective endogenous retroviruses can heavily influence the formation of the TCR repertoire reactive with exogenous retroviruses and determine the avidity of the response to retroviral infection. Given the overabundance of endogenous retroviruses in the human genome, these findings also suggest that endogenous retroviral proteins, presented by products of highly polymorphic *HLA* alleles, may shape the human TCR repertoire that reacts with exogenous retroviruses or other infecting pathogens, leading to interindividual heterogeneity.

## Author Summary

Our immune systems defend against viral infection. However, the immune response to a virus often differs substantially between individuals, as does the outcome of infection. The antiviral immune response relies on recognition of viral proteins by T lymphocytes using T cell antigen receptors (TCRs). TCRs are created randomly in an individual and each T lymphocyte has a different TCR. T lymphocytes with TCRs that recognize our own (self) proteins are removed during development, to prevent autoimmunity. Our cells can also make proteins that belong to endogenous retroviruses (ERVs), relics of ancestral retroviral infection that accumulated during evolution to constitute a large proportion of our genomes. The impact of ERVs on lymphocyte development and the subsequent influence on antiviral immunity is incompletely understood. Here, we use a mouse model to investigate this link and show that the T lymphocyte response to exogenous retrovirus infection is heavily influenced by an ERV. Surprisingly, we find that ERVs do not always have a negative impact on immunity, and in our model they improve the sensitivity with which T lymphocytes react to retroviral infection. This work may thus provide a basis for the understanding of the heterogeneity in immunity to retroviral infections in genetically diverse individuals.

## Introduction

Adaptive immunity to viral infection relies on appropriate activation of T cells by complexes of viral peptides with MHC molecules. The host *MHC* haplotype, the nature of the viral peptide targeted and the T cell receptor (TCR) repertoire of responding T cells are three interconnected parameters that play a decisive role in the outcome of infection. Indeed, the *MHC* is the predominant genetic factor affecting susceptibility to many infectious diseases [1]–[4]. For example, the *HLA* locus shows the strongest genetic association with control of HIV infection, with certain *HLA* alleles having been consistently found to confer a protective advantage [3], [5], [6]. Although the precise underlying mechanism is not completely understood, T cell responses restricted by protective *HLA*/*MHC* alleles often comprise narrower TCR repertoires, dominated by public TCR sequences, and exhibit higher magnitude, avidity or depth, and thus greater contribution to HIV or SIV control, than those restricted by non-protective *HLA*/*MHC* alleles [7]–[9].

Bias in the use of certain TCRVα (Vα) or TCRVβ (Vβ) chains has been frequently observed in the T cell response to several antigenic epitopes, and public T cell responses with identical or similar TCRs have been found to dominate the response of different individuals to a given epitope. Skewed TCR usage has often correlated with higher functional avidity to a given antigenic epitope, and, in diverse systems, also translated into more efficient protection against infection [10]–[12]. Despite the potential importance in cellular immunity to infection, however, the mechanisms by which TCR biases (and particularly high-avidity T cell responses to viral infections) are generated and maintained remains incompletely understood. The mechanisms leading to bias in the T cell response will vary considerably depending on the antigenic peptide and MHC combination. TCR repertoire bias can be generated during thymic selection, leaving only certain Vα or Vβ chains able to respond to a given antigen [10]. It can also be generated at the initiation of the immune response, where clones using particular Vα or Vβ chains may have a recruitment or proliferative advantage and can quickly dominate the response [10]. Lastly, bias can also be generated during chronic viral infection either due to preferential maintenance of certain T cell clones or differential margins for cross-reactivity with viral escape mutations [10] or by prior or concurrent infection with heterologous viruses, sharing cross-reactive epitopes [13].

We have previously described the TCRβ-transgenic strain EF4.1, which generates increased frequencies of CD4^+^ T cells reactive with the H2-A^b^-restricted env_122-141_ epitope of Friend murine leukemia virus (F-MLV) [14]. Virus-specific EF4.1 CD4^+^ T cells show bias in the use of endogenous Vα2 chains in their response to infection with Friend virus (FV), a retroviral complex of F-MLV and spleen focus-forming virus (SFFV) [14], [15]. Use of Vα2 chains by virus-specific CD4^+^ T cells creates higher avidity for the same epitope than use of other Vα chains, and although they represent a minority in the naïve repertoire, high-avidity Vα2 T cells become the dominant subset at the peak of the response [15]. Here we have examined the potential mechanisms underlying the formation of TCR repertoire diversity in this system, which might be responsible for the high-avidity response to FV infection. We have found that a thymic selection event was necessary for the dominance of Vα2 virus-specific CD4^+^ T cells during the response to FV infection. Selection of virus-specific CD4^+^ T cells was mediated by a self peptide encoded by an endogenous retrovirus with substantial similarity to F-MLV. Unexpectedly, despite deleting a sizeable fraction of virus-specific CD4^+^ T cells, negative selection by this endogenous retrovirus was necessary for a predominantly high-avidity response to FV infection.

## Results

### Higher functional avidity of Vα2 F-MLV env_122-141_-specific CD4^+^ T cells

On average, 4% of EF4.1 CD4^+^ T cells in virus-naïve mice react with the env_122-141_ peptide, of which approximately 25% stain positive with the anti-Vα2 monoclonal antibody B20.1 [14], [15]. Vα2 env-specific CD4^+^ T cells were previously [14], [15] found to be >30-fold more sensitive than non-Vα2 T cells to stimulation with a 20-mer env_122-141_ peptide spanning the core env_128-134_ epitope [16]. This higher avidity of Vα2 CD4^+^ T cells was not due to recognition of the core epitope-flanking residues by this family of Vα chains, as has been described for other TCR – epitope combinations [17], since it was maintained against a series of N-terminal truncated peptide epitopes (Figure S1A). Thus, Vα2 CD4^+^ T cells would recognize with higher avidity all the nested peptides of variable lengths likely to be generated during *in vivo* processing of env [18].

To examine whether the polyclonal Vα2 CD4^+^ T cell population displayed higher affinity for F-MLV env-derived epitopes even at the clonal level, we generated hybridoma cell lines from primary EF4.1 CD4^+^ T cells stimulated *in vitro* with either a low (10^−7^ M) or a high (10^−5^ M) peptide dose. In agreement with our previous findings [14], [15], 71% (20/28) and 30% (9/30) of hybridoma cell lines derived from CD4^+^ T cells stimulated with the low or high peptide dose, respectively, were Vα2^+^. Similarly to primary EF4.1 CD4^+^ T cells, randomly selected Vα2 T cell hybridomas were more sensitive to stimulation with all the peptides tested than non-Vα2 ones, irrespective of whether a high or low peptide dose was used for their generation (Figure S1B). Thus, the higher avidity of Vα2 CD4^+^ T cells for F-MLV env-derived epitopes was also observed at the level of individual clones.

To assess whether low-avidity F-MLV env-reactive CD4^+^ T cells were characterized by expression of any particular family of endogenous Vα chains, we screened env_122-141_-specific non-Vα2 CD4^+^ T cells for expression of *Trav* transcripts encoding different Vα families. Although this analysis indicated enrichment for *Trav9* expression (encoding Vα3), only a small percentage of env_122-141_-reactive non-Vα2 CD4^+^ T cells stained positive with the anti-Vα3.2 monoclonal antibody RR3-16 (*unpublished data*), and only 2 out of 4 F-MLV env-reactive non-Vα2 T cell hybridomas were positive for Vα3.2 (Table S1). However, Vα3.2 is used preferentially in CD8^+^ T cells in B6 mice, whereas the other three of the four expressed Vα3 family members are preferentially expressed in CD4^+^ T cells [19]. It was therefore possible that env_122-141_-reactive non-Vα2 CD4^+^ T cells that did not stain positive with the RR3-16 antibody were also expressing Vα3. Indeed, cloning and sequencing of expressed endogenous *Trav* genes from theses hybridomas revealed that they were all members of the *Trav9* family (Table S1). Thus, similarly to selective usage of Vα2 chains in high-avidity cells, low-avidity F-MLV env_122-141_-reactive CD4^+^ T cells selectively used Vα3 chains. However, in the absence of a Vα3-specific antibody that can detect all Vα3 family members, these cells were referred to here as non-Vα2 cells.

**Figure 1:**
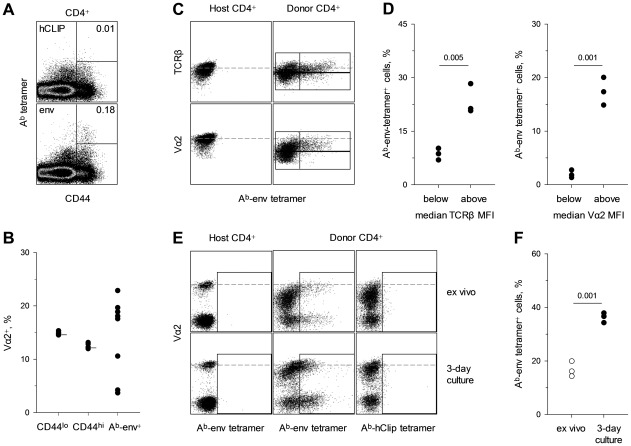
Detection of env-specific CD4^+^ T cells by A^b^-env_123-141_ tetramer eclipsed by antigen-induced TCR downregulation. (A) A^b^-hCLIP (control) or A^b^-env_123-141_ tetramer staining in total CD4^+^ T cells isolated from the spleen of wild-type B6 mice 7 days post FV infection. Plots are representative of 7 mice. (B) Frequency of Vα2 cells in either bulk naïve (CD44^lo^), bulk memory (CD44^hi^) or A^b^- env_123-141_ tetramer^+^ CD4^+^ T cells from the same FV infected mice. Horizontal short lines in naïve and memory subsets denote the mean frequency of Vα2 cells in the same populations from uninfected mice. Each symbol represents an individual mouse. (C–F) CD45.1^+^ EF4.1 CD4^+^ T cells were adoptively transferred into wild-type B6 recipients that were infected with FV the same day. (C) A^b^-env_123-141_ tetramer staining in host (CD45.1^−^) or donor (CD45.1^+^) CD4^+^ T cells according to TCRα or TCRβ staining. Gates in donor CD4^+^ T cells are set around the median TCRα and TCRβ staining, respectively. (D) Percentage of A^b^-env_123-141_ tetramer^+^ cells in donor CD4^+^ T cells with TCRβ (*left*) or TCRα (*right*) staining below or above the median. (E) A^b^-hCLIP or A^b^-env_123-141_ tetramer staining in host or donor CD4^+^ T cells from the same recipients assessed directly *ex vivo* (*top*) or following 3-day *in vitro* culture in the absence of antigenic stimulation (*bottom*). (F) Percentage of A^b^-env_123-141_ tetramer^+^ cells in donor CD4^+^ T cells before and after *in vitro* culture. In (D) and (F) each symbol represents an individual mouse from one of two experiments.

Lastly, we tested whether biased use of Vα2 chains also characterized the response of non-transgenic CD4^+^ T cells to F-MLV env. CD4^+^ T cells from wild-type (wt) C57BL/6 (B6) mice 7 days post FV infection were stained with an env_123-141_-presenting tetramer (A^b^-env_123-141_). In comparison with a control tetramer, staining with A^b^-env_123-141_ tetramer identified a measurable population of env_122-141_-specific CD4^+^ T cells in all infected mice (Figure 1A), in agreement with published data [20], [21]. FV infection had no impact on the frequency of Vα2 cells in naïve (CD44^lo^) and total memory (CD44^hi^) CD4^+^ T cells (15% and 12%, respectively), with minimal variation between individual mice (Figure 1B). In contrast, the frequency of Vα2 cells in A^b^-env_123-141_ tetramer^+^ CD4^+^ T cells varied considerably between 4% and 23%. These results revealed substantial deviation in Vα2 usage in A^b^-env_123-141_ tetramer^+^ CD4^+^ T cells from the same usage in total CD4^+^ T cells, but also indicated substantial heterogeneity. However, this particular tetramer is known to bind only some env_122-141_-specific T cell clones but not others [14], [22]. Furthermore, at the peak of their response, env_122-141_-specific CD4^+^ T cell reversibly downregulate up to 70% of their surface TCR [15], [23], which could prevent tetramer binding. Indeed, combining adoptive transfer of EF4.1 CD4^+^ T cells and tetramer staining revealed that A^b^-env_123-141_ tetramer staining was restricted to env_122-141_-reactive CD4^+^ T cells with above-average TCR levels, independently of Vα usage, and TCR re-expression improved tetramer staining (Figure 1C–F). Thus, detection of env_122-141_-reactive CD4^+^ T cells by A^b^-env_123-141_ tetramer staining was eclipsed by *in vivo* antigen-induced TCR downregulation. Collectively, these results both validated and necessitated the use of env_122-141_-reactive EF4.1 CD4^+^ T cells that can be indelibly identified, independently of A^b^-env_123-141_ tetramer binding, to study the requirements for induction of a high-avidity CD4^+^ T cell response to F-MLV env.

### Deletion of F-MLV env_122-141_-specific CD4^+^ T cells by *Emv2*-encoded env

F-MLV env_122-141_-reactive CD4^+^ T cells in EF4.1 mice have a naïve phenotype [14], and it was therefore likely that the F-MLV env_122-141_-reactive TCR repertoire and associated avidity differences were the result of thymic selection events. We searched the mouse proteome for the presence of self-derived epitopes with homology to F-MLV env_122-141_. This approach identified the single-copy endogenous ecotropic MLV at the *Emv2* locus [24]. *Emv2* shares 80% homology with F-MLV at the DNA sequence level, and although it represents a full-length provirus, it is unable to produce infectious particles due to a single inactivating point-mutation in the *pol* gene [24]. Nevertheless, *Emv2* has full potential for *env* expression, and, importantly, the env_122-141_ epitope is almost identical between *Emv2* and F-MLV, with the exception of a Y instead of an L at position 128 (Figure S2A). For this reason, *Emv2* and F-MLV env-derived epitopes were referred to here as env_122-141_Y and env_122-141_L, respectively. Position 128, together with 129 and 133, have been previously mapped as important contact residues for the SB14-31 TCR (Figure S2B), which was the donor of the TCRβ chain transgene used in EF4.1 mice [16].

**Figure 2:**
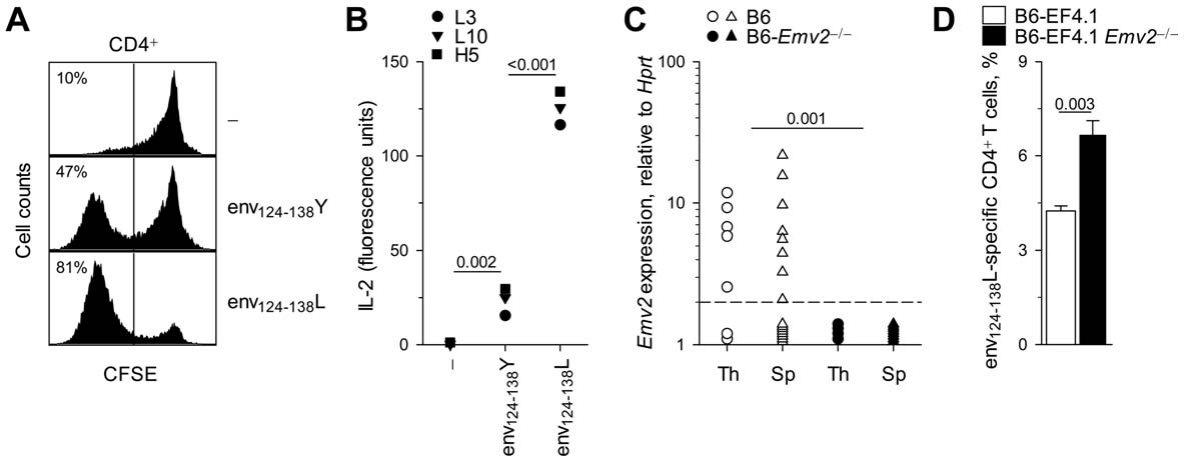
*Emv2* selects against a fraction of env_124-138_L-specific CD4^+^ T cells. (A) Dilution of prior CFSE label by primary EF4.1 CD4^+^ T cells incubated for three days *in vitro* in the absence of peptide stimulation (-) or in the presence of 10^−5^ M env_124-138_L or env_124-138_Y peptides. Numbers within the plots denote the percentage of CFSE^−^ cells and are representative of 4 mice per condition. (B) IL-2 production by three env_124-138_L-specific hybridoma T cell lines in response to *in vitro* stimulation with the same peptides at 5×10^−6^ M. (C) *Emv2* transcription, relative to *Hprt* transcription, in thymi (Th) and spleens (Sp) of wild-type B6 or *Emv2*-deficient B6 mice (B6-*Emv2* ^−/−^). Each symbol is an individual mouse. The dashed line represents the limit of detection. (D) Frequency of env_124-138_L-specific cells in primary CD4^+^ T cells from B6 (B6-EF4.1) or *Emv2*-deficient B6 (B6-EF4.1 *Emv2* ^−/−^) EF4.1 mice. Data are the means ± SEM (*n* = 9) from 3 experiments.

We next investigated whether or not *Emv2* could be involved in T cell selection. *In vitro* stimulation with the env_124-138_Y epitope activated a fraction of EF4.1 CD4^+^ T cells, which was however smaller than the fraction activated by the env_124-138_L epitope (Figure 2A). As EF4.1 mice generate a polyclonal TCR repertoire, it was unclear whether the same CD4^+^ T cells could respond to both epitopes. However, analysis of env_124-138_L-reactive T cell hybridomas revealed the same TCR could be activated by both env_124-138_L and env_124-138_Y epitopes, albeit less potently by the latter peptide (Figure 2B). Thus, F-MLV env_124-138_-reactive TCRs have the potential to recognize *Emv2* env. This analysis also revealed that *Emv2* was not causing complete tolerance of either env_124-138_L or env_124-138_Y epitopes. We then confirmed that *Emv2* was expressed in primary and secondary lymphoid organs. Using primers specific to the spliced *env* mRNA that could distinguish between genuine transcripts and contaminating genomic DNA, *Emv2* was found to be expressed at low levels in the majority of mice tested (Figure 2C). This low level of expression was further confirmed by comparison with a newly-generated B6 congenic strain lacking *Emv2* (Figure S3). To evaluate the extent of *Emv2*-mediated deletion of env-reactive CD4^+^ T cells more directly, we generated B6-*Emv2* ^−/−^ EF4.1 mice and compared them with *Emv2*-expressing EF4.1 mice. *Emv2*-deficient EF4.1 mice contained a significantly higher frequency of env_124-138_L-reactive CD4^+^ T cells than *Emv2*-sufficient EF4.1 mice, with *Emv2*, when present, being responsible for the deletion of approximately 35% of these cells (Figure 2D). Thus, albeit low, expression of *Emv2* in B6 mice significantly impacted on the frequency of env_124-138_L-reactive EF4.1 CD4^+^ T cells.

### The *Emv2*-selected CD4^+^ T cell repertoire retains full antiviral activity

**Figure 3:**
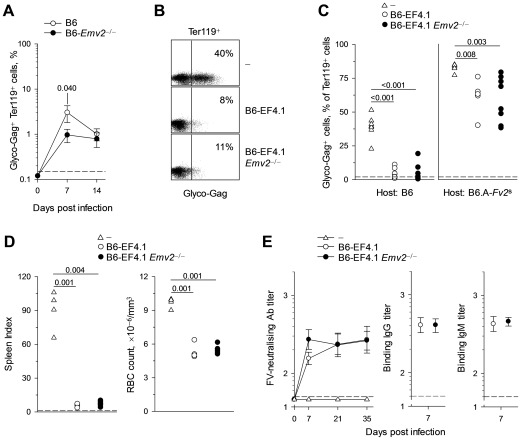
*Emv2*-selected CD4^+^ T cells retain full antiviral activity. (A) Mean frequency (± SEM, *n* = 8–19) of FV-infected (glyco-Gag^+^) Ter119^+^ cells in the spleens of FV-infected B6 or *Emv2*-deficient B6 mice (B6-*Emv2* ^−/−^). (B–C) CD4^+^ T cells isolated from either B6 (B6-EF4.1) or *Emv2*-deficient B6 (B6-EF4.1 *Emv2* ^−/−^) EF4.1 mice were adoptively transferred into B6 or B6.A-*Fv2* ^s^ recipients that were infected with FV the same day and analyzed 7 days later. (B) Flow cytometric example of FV-infected Ter119^+^ cells from B6 recipients and (C) frequency of FV-infected cells in Ter119^+^ cells from the spleens of B6 or B6.A-*Fv2* ^s^ recipients of CD4^+^ T cells. Control B6 and B6.A-*Fv2* ^s^ mice that received no CD4^+^ T cells (-) are also included. Each symbol is an individual mouse. (D) Spleen index (*left*) and RBC count (*right*) of B6-*Rag1* ^−/−^ *Fv2* ^s^ mice that were infected with FV and either received the same day CD4^+^ T cells isolated from either B6 (B6-EF4.1) or *Emv2*-deficient B6 (B6-EF4.1 *Emv2* ^−/−^) EF4.1 mice or no cells (-). Each symbol is an individual mouse analyzed 3 weeks post infection. (E) Titers of FV-neutralizing antibodies during the course of FV infection (*left*) and titers of F-MLV-infected cell-binding IgG (*middle*) and IgM (*right*) 7 days post FV infection, in the sera of B6-*Tcra* ^−/−^ mice that either received CD4^+^ T cells isolated from either B6 (B6-EF4.1) or *Emv2*-deficient B6 (B6-EF4.1 *Emv2* ^−/−^) EF4.1 mice or no cells (-) the day of the infection. Dashed lines represent the limit of detection. Data are the means ± SEM (*n* = 11–12) from 2 experiments.

*Emv2*-mediated deletion of a proportion of env_124-138_L-reactive EF4.1 CD4^+^ T cells suggested that *Emv2* may impinge on resistance to FV infection. We therefore examined the possible effect of *Emv2* expression on FV control. Firstly, we infected non-transgenic B6 and *Emv2*-deficient B6 mice and measured the levels of infected cells in the spleen. B6 mice are relatively resistant to FV infection due to genetic resistance at the *Fv2* locus and due to mounting a strong adaptive immune response [1], [25], resulting in control of the infection by the third week. Percentages of FV-infected (glyco-Gag^+^) erythroid precursor (nucleated Ter119^+^) cells were significantly lower in B6-*Emv2* ^−/−^ mice than in wt counterparts at day 7 of infection (Figure 3A). Nevertheless, wt B6 mice effectively controlled FV infection to levels comparable with those in B6-*Emv2* ^−/−^ mice by the second week of infection (Figure 3A). Thus, *Emv2* deficiency did not extensively increase the natural resistance of B6 mice to FV infection.

The modest increase in resistance to FV infection in B6-*Emv2* ^−/−^ mice suggested that this low *Emv2* expression was immunologically relevant, but did not indicate if any arm of the adaptive immune response was affected. We thus measured the FV-specific CD4^+^ T cell, CD8^+^ T cell and antibody responses in these mice. In contrast to the MHC class II-restricted env_122-141_L epitope, the FV-derived MHC class I-restricted epitopes that have been described do not share extensive homology or cross-reactivity with those derived from *Emv2* [26]–[28]. We examined the CD8^+^ T cell response to FV by measuring numbers of activated CD44^hi^CD43^+^CD8^+^ T cells, irrespective of antigen specificity, in the spleens of B6 and B6-*Emv2* ^−/−^ mice 7 days post FV infection (Figure S4A). The two types of host showed comparable expansion of CD44^hi^CD43^+^CD8^+^ T cells, suggesting they mounted a CD8^+^ T cell response of similar magnitude (Figure S4A). We further measured the CD8^+^ T cell response to the immunodominant D^b^-restricted epitope from the leader sequence gPr80gag_85-93_ encoded by the F-MuLV *gag* gene [28]. CD8^+^ T cells specific to the gPr80gag_85-93_ epitope display strong bias for the use of Vα3.2 and Vβ5.2 in combination, which allows their identification by flow cytometry [29]. Expectedly, FV infection led to an increase in the percentage of Vα3.2^+^Vβ5.2^+^ cells in antigen-experienced (CD44^hi^), but not naïve (CD44^lo^) CD8^+^ T cells (Figure S4B). However, this expansion of Vα3.2^+^Vβ5.2^+^CD44^hi^CD8^+^ T cells was comparable in B6 and B6-*Emv2* ^−/−^ mice 7 days post FV infection (Figure S4B).

We next examined the FV-specific antibody response of B6 and B6-*Emv2* ^−/−^ hosts. As FV-neutralizing antibodies are not readily detected in B6 mice on day 7 post FV infection [25], we measured titers of antibodies that were able to bind F-MLV-infected cells (Figure S4C). On day 7 post FV infection all mice produced both IgG and IgM F-MLV-infected cell-binding antibodies that could be measured by flow cytometry (*unpublished data*). However, titers of these antibodies were low at this early time-point and in a proportion of FV-infected mice they were below 50, a value that we set as the detection limit (Figure S4C). Importantly, serum titers of both IgG and IgM F-MLV-infected cell-binding antibodies were similar between B6 and B6-*Emv2* ^−/−^ hosts (Figure S4C).

Lastly, the frequency of A^b^-env_123-141_ tetramer^+^ CD4^+^ T cells as well as the frequency of Vα2 cells within this population was highly variable between individual mice and as a result not statistically different between groups of B6 and B6-*Emv2* ^−/−^ hosts on day 7 post FV infection (Figure S4D). However, staining with the A^b^-env_123-141_ tetramer may have underestimated the frequency of env_122-141_L-reactive CD4^+^ T cells (Figure 1) and it was also possible that env_122-141_L-reactive CD4^+^ T cells selected in the presence or absence of *Emv2* expressed TCRs with distinct A^b^-env_123-141_ tetramer-binding properties. Furthermore, virus-specific CD4^+^ T cells can mediate both direct and indirect protection against FV infection [15], [23], [30], and env_122-141_-specific CD4^+^ T cells have been shown to mediate direct cytotoxic activity [31]. It was thus uncertain whether weakened immunity in *Emv2*-expressing mice was directly linked to a potentially less effective CD4^+^ T cell response. We therefore examined the effect of *Emv2* expression on the FV-specific CD4^+^ T cell response functionally and directly. To this end, equal numbers of *Emv2*-selected or -nonselected EF4.1 CD4^+^ T cells were transferred into the same type of host. This approach ensured that only donor EF4.1 CD4^+^ T cells differed with respect to exposure to *Emv2*. Surprisingly, the two types of donor CD4^+^ T cells provided comparable and almost complete protection of wild-type B6 hosts, at the peak of FV replication on day 7 post infection (Figure 3B, C). To rule out that differences in antiviral activity between the two types of donor CD4^+^ T cells were not missed because this activity was already maximal, we have additionally used B6.A-*Fv2^s^* hosts, expressing the susceptibility allele at the *Fv2* locus, which confers susceptibility to FV infection by enhancing proliferation of infected erythroid precursors [25]. The two types of donor CD4^+^ T cells provided significant, suboptimal and, importantly, comparable protection in B6.A-*Fv2^s^* hosts, at the peak of FV replication and expansion of infected erythroid precursors on day 7 post infection in this strain (Figure 3C). Thus, *Emv2*-mediated selection did not impair the antiviral activity of CD4^+^ T cells exerted in wt hosts. To further examine direct CD4^+^ T cell-mediated protection we transferred equal numbers of *Emv2*-selected or -nonselected EF4.1 CD4^+^ T cells into T and B cell-deficient *Rag1* ^−/−^ *Fv2* ^s^ hosts. Both types of donor CD4^+^ T cells were similarly protective against severe FV-induced splenomegaly (Figure 3D) that otherwise develops in these hosts [15]. In addition, the two types of donor CD4^+^ T cells caused comparable levels of anemia in these T and B cell -deficient hosts (Figure 3D), which results from bone marrow pathology [14]. Lastly, FV-neutralizing antibodies were similarly and efficiently induced in T cell-deficient *Tcra* ^−/−^ hosts by transfer of either type of donor CD4^+^ T cells, although they were slightly, but not significantly higher in hosts of *Emv2*-nonselected CD4^+^ T cells on day 7 post infection (Figure 3E). Nevertheless, at this time-point, the two types of donor CD4^+^ T cells induced comparable titers of IgG or IgM antibodies that were able to bind F-MLV-infected cells, which also included antibodies potentially mediating antibody-dependent cell-mediated cytotoxicity (Figure 3E). Collectively, these results demonstrated that despite selecting against a significant fraction of env_124-138_L-reactive CD4^+^ T cells, *Emv2* expression did not compromise CD4^+^ T cell function against FV infection.

### Selection by *Emv2* promotes a higher-avidity response to F-MLV

**Figure 4:**
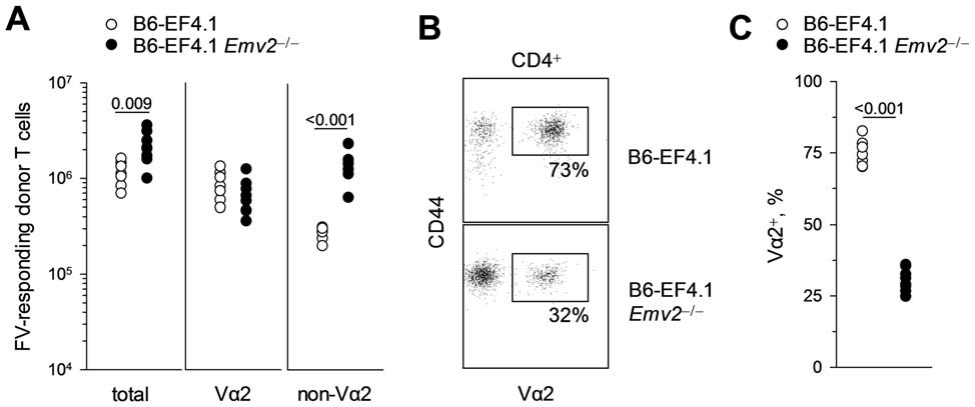
*Emv2*-selected CD4^+^ T cells mount a predominantly high-avidity response. (A–C) CD45.2^+^ (*Ptprc* ^2/2^) CD4^+^ T cells isolated from either B6 (B6-EF4.1) or *Emv2*-deficient B6 (B6-EF4.1 *Emv2* ^−/−^) EF4.1 donor mice were adoptively transferred into *Ptprc* ^1/2^ B6 recipients that were infected with FV the same day and analyzed 7 days later. (A) Absolute number of total, Vα2 or non-Vα2 FV-responding (CD44^hi^) donor (CD45.2^+^CD45.1^−^) CD4^+^ T cells isolated from the spleens of recipient mice according to donor type. (B) Flow cytometric example and (C) frequency of high-avidity Vα2 cells in responding CD4^+^ T cells according to donor type. In (A) and (C) each symbol is an individual mouse.

Retention of full CD4^+^ T cell-mediated antiviral activity, despite deletion of over a third of env_124-138_L-reactive CD4^+^ T cells in *Emv2*-expressing mice, suggested that the deleted cells were not contributing to immunity against FV infection. We therefore assessed the impact of *Emv2* expression on both the magnitude and composition of the CD4^+^ T cell response to FV. Equal numbers of EF4.1 CD4^+^ T cells from either *Emv2*-sufficient or -deficient donor B6 mice, positive for CD45.2 (encoded by the *Ptprc* ^2^ allele), were adoptively transferred into *Ptprc* ^1/2^ syngeneic B6 recipients that were positive for both CD45.1 and CD45.2. Recipient mice were infected with FV on the day of T cell transfer and FV-responding donor CD4^+^ T cells were identified as CD44^hi^CD45.2^+^CD45.1^−^ cells (Figure S5). Consistent with increased precursor frequency in *Emv2*-deficient donor mice, significantly higher numbers of total responding CD4^+^ T cells could be recovered at the peak of the response from secondary recipients that received *Emv2*-nonselected than those that received *Emv2*-selected donor CD4^+^ T cells (Figure 4A). Notably, the two types of donor CD4^+^ T cells generated comparable numbers of high-avidity responding CD4^+^ T cells, and the numerical increase in total numbers of responding CD4^+^ T cells from *Emv2*-nonselected donors was due to significantly higher expansion of low-avidity non-Vα2 responding CD4^+^ T cells from these donors in comparison with the expansion of non-Vα2 responding CD4^+^ T cells from *Emv2*-selected donors (Figure 4A). As a result, peak expansion of *Emv2*-selected CD4^+^ T cells was dominated by high-avidity Vα2 CD4^+^ T cells, whereas that of *Emv2*-nonselected CD4^+^ T cells was dominated by low-avidity non-Vα2 CD4^+^ T cells (Figure 4B, C). Thus, *Emv2* expression converted a predominantly low-avidity response to FV to a predominantly high-avidity response.

### *Emv2*-encoded env preferentially deletes non-Vα2 CD4^+^ T cells

**Figure 5:**
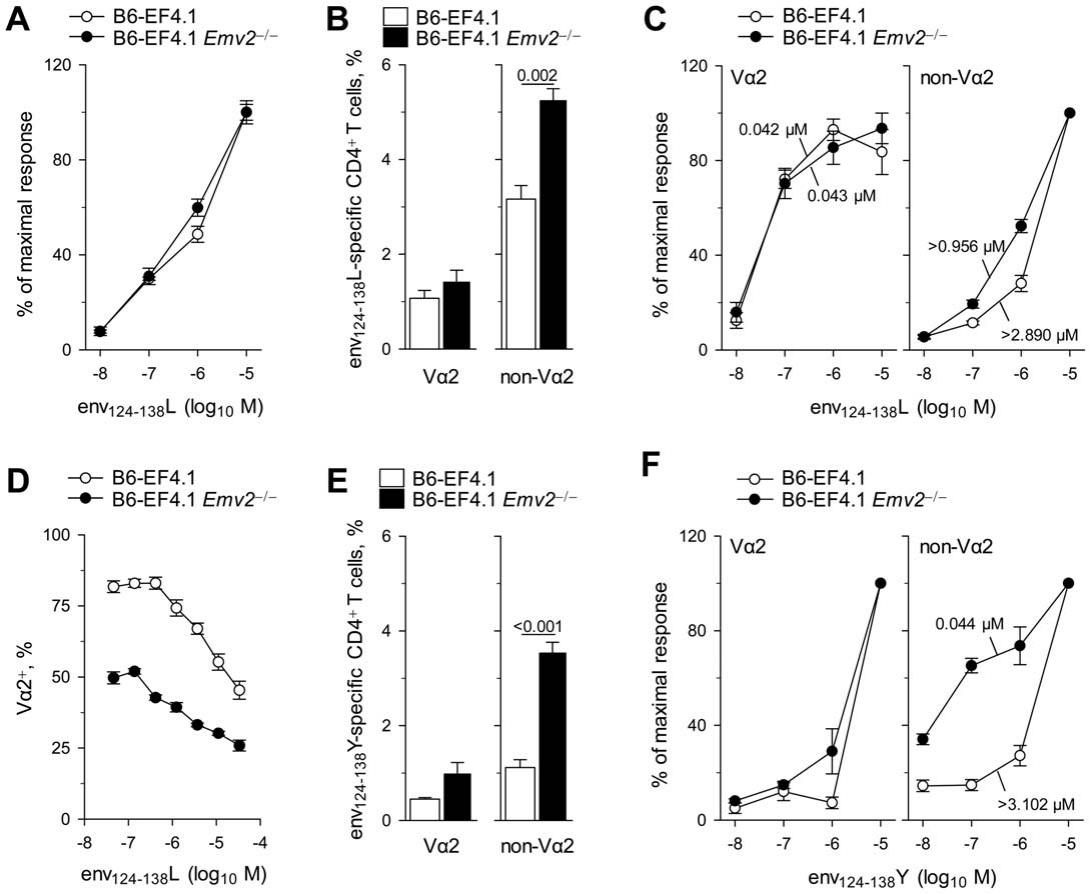
*Emv2* preferentially selects against non-Vα2 env-specific CD4^+^ T cells. (A) Dose-response to env_124-138_L stimulation of CD4^+^ T cells isolated from either B6 (B6-EF4.1) or *Emv2*-deficient B6 (B6-EF4.1 *Emv2* ^−/−^) EF4.1 mice. (B) Frequency of env_124-138_L-specific cells in Vα2 or non-Vα2 primary CD4^+^ T cells from the same donors. (C) Functional avidity of *Emv2*-selected (B6-EF4.1) or -nonselected (B6-EF4.1 *Emv2* ^−/−^) EF4.1 CD4^+^ T cells for env_124-138_L. (D) Frequency of Vα2 cells in env_124-138_L-specific CD4^+^ T cells from the same donors as a function of peptide concentration. (E) Frequency of env_124-138_Y-specific cells in Vα2 or non-Vα2 primary CD4^+^ T cells from the same donors. (F) Functional avidity of *Emv2*-selected (B6-EF4.1) or -nonselected (B6-EF4.1 *Emv2* ^−/−^) EF4.1 CD4^+^ T cells for env_124-138_Y. Numbers in (C) and (F) represent the ED_50_. Data in (A–F) are the means ± SEM (*n* = 9–12) of 18-hr stimulations from 3 experiments.

The shift from a predominantly Vα2 response of *Emv2*-selected CD4^+^ T cells to a predominantly non-Vα2 response of *Emv2*-nonselected CD4^+^ T cells could be the result of *Emv2*-induced modulation of either the relative frequency in the naïve repertoire of the two subsets of env_124-138_L-reactive CD4^+^ T cells, or their relative avidity for env_124-138_L (or both). We first measured the overall functional avidity to env_124-138_L of EF4.1 CD4^+^ T cells selected with or without *Emv2* as an indicator of potential avidity repertoire changes. Surprisingly, although the presence of *Emv2* reduced the precursor frequency of env_124-138_L-reactive CD4^+^ T cells, it had no effect on the avidity with which they responded to env_124-138_L stimulation (Figure 5A). This result suggested that *Emv2*-mediated selection either affected high- and low-avidity cells similarly, or that potential loss of higher-avidity T cells was compensated by an increase in average avidity of the remaining T cells. To examine whether *Emv2* preferentially selected against high-avidity env_124-138_L-reactive cells, we measured their frequency separately in either Vα2 or non-Vα2 CD4^+^ T cells from EF4.1 mice. Notably, *Emv2* expression significantly reduced the frequency of non-Vα2, but not Vα2 env_124-138_L-reactive cells in EF4.1 CD4^+^ T cells (Figure 5B), indicating that it only selected against non-Vα2 CD4^+^ T cells. Correspondingly, the avidity of Vα2 CD4^+^ T cells to env_124-138_L was not altered by *Emv2* expression, whereas the avidity of non-Vα2 CD4^+^ T cells was 3-fold higher in the absence than in the presence of *Emv2* (Figure 5C). Nevertheless, non-Vα2 CD4^+^ T cells from *Emv2*-deficienct mice still displayed lower avidity than Vα2 CD4^+^ T cells from either *Emv2*-deficienct or -sufficient mice (Figure 5C).

To test whether *Emv2*-mediated changes in the frequency and avidity for env_124-138_L of non-Vα2 CD4^+^ T cells could account for the dominance of this subset in the *in vivo* response to FV of *Emv2*-nonselected CD4^+^ T cells, we examined the *in vitro* response of *Emv2*-selected or -nonselected primary naïve EF4.1 CD4^+^ T cells to env_124-138_L stimulation. As a result of differences in initial frequency and functional avidity between virus-naïve Vα2 and non-Vα2 env_122-141_-specific cells, the composition of the responding population varied according to the amount of env_122-141_ presentation [14] and Vα2 T cells dominated the response at doses lower than 10^−7^ M (Figure 5D). Importantly, this percentage of Vα2 cells was significantly lower at all peptide doses in CD4^+^ T cells selected in the absence than in the presence of *Emv2* (Figure 5D), demonstrating that selection by this single provirus heavily influenced the clonal composition of env_124-138_L-reactive CD4^+^ T cells, in favor of high-avidity cells. We further confirmed that this effect of *Emv2* expression of reducing the overall frequency of env_124-138_L-reactive cells, but significantly increasing the percentage of high-avidity Vα2 cells in the env_124-138_L-reactive population was already evident in CD4^+^CD8^−^ thymocytes (Figure S6), consistent with a thymic, rather than peripheral event.

Preferential deletion by *Emv2* of non-Vα2 CD4^+^ T cells, which had low avidity for F-MLV env_124-138_L raised the possibility that these cells may have been cross-reactive with *Emv2*-encoded env_124-138_Y. Indeed, lack of *Emv2* expression in EF4.1 mice had a small, non-significant effect on env_124-138_Y-reactive Vα2 CD4^+^ T cells, but caused a significant 3.5-fold increase in the frequency of env_124-138_Y-reactive non-Vα2 CD4^+^ T cells (Figure 5E). Furthermore, Vα2 CD4^+^ T cells from either *Emv2*-deficient or -sufficient EF4.1 mice could only react with env_124-138_Y at the highest dose of 10^−5^ M, whereas non-Vα2 CD4^+^ T cells from *Emv2*- deficient mice were markedly more sensitive to env_124-138_Y than those from *Emv2*-sufficient mice (and as sensitive as Vα2 CD4^+^ T cells to env_124-138_L) (Figure 5F). Together, these findings indicated that *Emv2* expression was not affecting env_124-138_L-reactive Vα2 CD4^+^ T cells because they displayed low avidity for env_124-138_Y, but was deleting a significant proportion of non-Vα2 CD4^+^ T cells that could react with either env_124-138_L or env_124-138_Y.

### Shaping of env-reactive CD4^+^ T cell repertoire depth by *Emv2*

**Figure 6:**
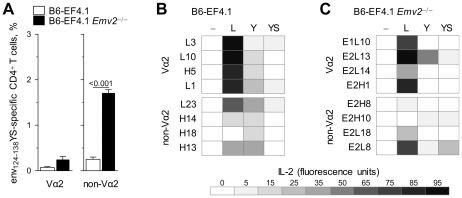
Cross-reactivity of individual Vα2 or non-Vα2 CD4^+^ T cells. (A) Frequency of env_124-138_YS-reactive cells in Vα2 or non-Vα2 CD4^+^ T cells isolated from either B6 (B6-EF4.1) or *Emv2*-deficient B6 (B6-EF4.1 *Emv2* ^−/−^) EF4.1 mice. Data are the means ± SEM (*n* = 9) of 18-hr stimulations from 3 experiments. (B–C) IL-2 production in response to stimulation with 5×10^−6^ M env_124-138_L (L), env_124-138_Y (Y) or env_124-138_YS (YS) in comparison with the absence of peptide stimulation (-) of Vα2 or non-Vα2 env_124-138_L-reactive hybridoma T cell lines derived from *Emv2* ^+/+^ (B) or *Emv2* ^−/−^ (C) EF4.1 mice.

Although EF4.1 CD4^+^ T cells selected by *Emv2* mounted high-avidity responses to the index env_124-138_L sequence *in vitro*, and to FV infection *in vivo*, and retained full antiviral activity, counter-selection of env_124-138_Y-reactive clones indicated that this repertoire would be less able to respond to viral escape mutations, and especially to an L128Y mutation. To extend these findings, we used another variant of env, which differed from F-MLV env in two of the three putative TCR-binding residues. This variant has Y and S in positions 128 and 129, respectively (referred to as env_124-138_YS) and is a naturally-occurring functional form of ecotropic env, encoded by the *Fv4* locus in certain strains and species of mouse, other than the B6 strain [32], [33]. Again, a very small fraction of EF4.1 Vα2 CD4^+^ T cells could react to env_124-138_YS, regardless of the presence or absence of *Emv2* (Figure 6A). In contrast, lack of *Emv2* led to a 7-fold increase in the frequency of env_124-138_YS-reactive EF4.1 non-Vα2 CD4^+^ T cells, which now made a sizable fraction (Figure 6A). Thus, non-Vα2 CD4^+^ T cells from *Emv2*-deficient EF4.1 mice could react with the index sequence and the two env variants and with high avidity to env_124-138_Y, suggesting that *Emv2*-mediated selection significantly reduced the ability of CD4^+^ T cells, at the population level, to recognize these env variants.

This analysis of polyclonal cells from *Emv2*-deficient EF4.1 mice did not reveal whether the same T cell could react to all three env variants or if env_124-138_L-, env_124-138_Y- and env_124-138_YS-reactive non-Vα2 CD4^+^ T cells were distinct. We therefore tested the reactivity of hybridoma cell lines generated from env_124-138_L-reactive EF4.1 CD4^+^ T cells that developed either in the presence or the absence of *Emv2* expression to other env variants. Similarly to non-Vα2 CD4^+^ T cell hybridomas from *Emv2*-sufficient donors, all 4 non-Vα2 CD4^+^ T cell hybridomas tested from *Emv2*-deficient donors used members of the TCRVα3 family (encoded by the *Trav9* gene family; Table S2). Notably, neither *Emv2*-selected nor -nonselected env_124-138_L-reactive non-Vα2 CD4^+^ T cell hybridomas responded to env_124-138_Y more potently than Vα2 CD4^+^ T cell hybridomas from the same donor strain, and only 1 out of 4 had a measureable response to env_124-138_YS (Figure 6B, C). These findings suggested that the env_124-138_L-reactive non-Vα2 CD4^+^ T cells that developed in *Emv2*-deficient EF4.1 mice were largely distinct from env_124-138_Y- and env_124-138_YS-reactive T cells in the same mice. They also indicated that env_124-138_L-reactive non-Vα2 CD4^+^ T cells were not inherently more cross-reactive than env_124-138_L-reactive Vα2 CD4^+^ T cells at the clonal level.

**Figure 7:**
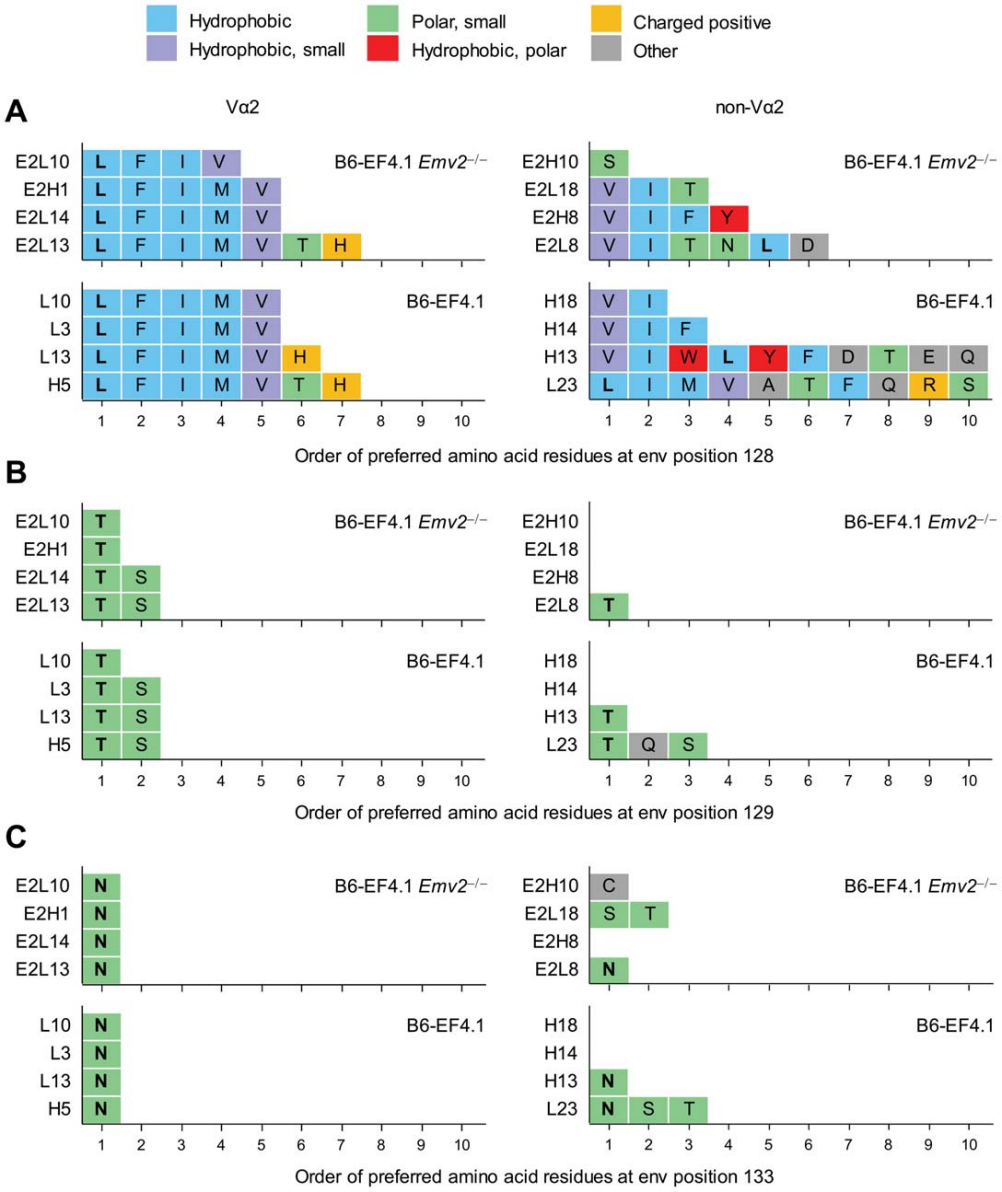
Depth of Vα2 or non-Vα2 env-specific CD4^+^ T cell repertoires. (A–C) Vα2 or non-Vα env_124-138_L-reactive hybridoma T cell lines were derived from *Emv2* ^+/+^ (B6-EF4.1) or *Emv2* ^−/−^ (B6-EF4.1 *Emv2* ^−/−^) EF4.1 mice and tested for reactivity against a library of env_126-138_ peptide epitopes. The amino acid residues in positions 128 (A), 129 (B) and 133 (C) that elicited at least 40% of the maximal response are listed in the order of preference by the individual clones.

To gain a more detailed view of the depth, defined here as the ability to tolerate epitope mutations, of env_124-138_L-reactive Vα2 or non-Vα2 TCRs, we screened *Emv2*-selected or -nonselected env_124-138_L-reactive T cell hybridomas for reactivity against a library of env_126-138_ peptide variants carrying all possible single mutations in each of the amino acid residues in positions 128, 129 and 133 (Figure S7). Amino acids that elicited at least 40% of the maximal response were listed in the order they were preferred by the individual TCRs (Figure 7). All Vα2 T cell hybridomas displayed strong preference for L at position 128 and also recognized similar amino acids with hydrophobic side chains, namely F, I, M and V, but not the less hydrophobic Y (Figure 7A). Vα2 T cell hybridomas also showed strong preference and specificity for the amino acid residues of the index sequence against which they were derived, T or highly similar S at position 129, and N at position 133 (Figure 7B, C). Overall, the depth of Vα2 T cell hybridomas was highly homogeneous and unaffected by *Emv2* expression. In contrast to Vα2 T cell hybridomas, and as expected by their low avidity for the index env_124-138_L sequence, none of the non-Vα2 T cell hybridomas derived from *Emv2*-deficient mice displayed strong preference for L at position 128 (Figure 7A). The latter hybridomas did, however, respond strongly to env variants with a different amino acid residue at this position, most frequently V or I, or in the case of clone E2H10 the unrelated S (Figure 7A). Non-Vα2 T cell hybridomas selected by *Emv2* were also heterogeneous, with two clones showing similar preference and specificity for V or I, and two other clones showing much wider reactivity to at least 10 different amino acid residues, including L (Figure 7A). Furthermore, the low reactivity to the index env_124-138_L sequence of two of the four non-Vα2 T cell hybridomas derived from *Emv2*-deficient mice, but not those derived from *Emv2*-sufficient mice, could be enhanced by substitutions at another position (C for clone E2H10, and S or T for clone E2L18, instead of N at position 133) (Figure 7C). Non-Vα2 T cell hybridomas that could recognize L at position 128 also preferred the amino acid residue of the index env_124-138_L sequence at the two other positions (T and N for positions 129 and 133, respectively) (Figure 7B, C). Collectively, these results confirmed the differential preference for L at position 128 between Vα2 and non-Vα2 T cell hybridomas and further suggested that selection by *Emv2* enriched the non-Vα2 repertoire for clones with relative indifference for this position.

### Genetic contribution to a high-avidity env_124-138_L-reactive CD4^+^ T cell repertoire

**Figure 8:**
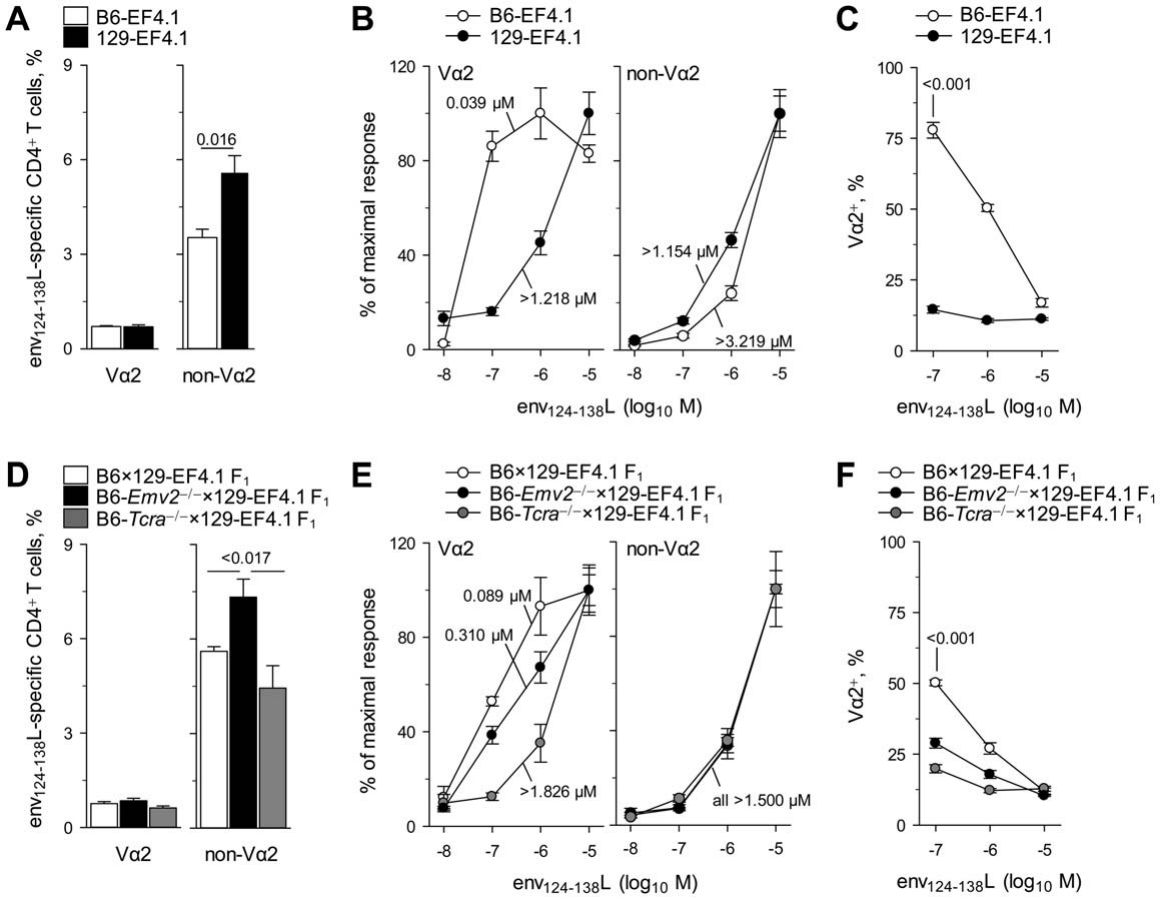
Genetic contribution to a high-avidity env-reactive CD4^+^ T cell repertoire. (A) Frequency of env_124-138_L- reactive cells in Vα2 or non-Vα2 primary CD4^+^ T cells isolated from either B6 (B6-EF4.1) or 129S8 (129S8-EF4.1) EF4.1 mice. (B) Functional avidity of env_124-138_L-reactive Vα2 or non-Vα2 primary CD4^+^ T cells from the same donors in A. (C) Frequency of Vα2 cells in env_124-138_L-reactive CD4^+^ T cells from the same donors in A as a function of peptide concentration. (D) Frequency of env_124-138_L- reactive cells in Vα2 or non-Vα2 primary CD4^+^ T cells isolated from either B6×129S8-EF4.1 F_1_, B6-*Emv2* ^−/−^×129S8-EF4.1 F_1_ or B6-*Tcra* ^−/−^×129S8-EF4.1 F_1_, EF4.1 mice. (E) Functional avidity of env_124-138_L-reactive Vα2 or non-Vα2 primary CD4^+^ T cells from the same donors in D. (F) Frequency of Vα2 cells in env_124-138_L-reactive CD4^+^ T cells from the same donors in D as a function of peptide concentration. Numbers in (B) and (E) represent the ED_50_. In (C) and (F) the CD4^+^ T cell response elicited by the last peptide dose (10^−8^ M) was too small to allow accurate measurement of the frequency of Va2 cells and was therefore omitted. Data in (A–F) are the means ± SEM (*n* = 4–8) of 18-hr stimulations from 3 experiments.

Analysis of the env-reactive CD4^+^ T cell repertoire in B6 mice revealed a clear effect of *Emv2*-mediated selection. However, in addition to *Emv2*, the presence of numerous other endogenous retroviruses could affect the formation of the env_124-138_L-reactive CD4^+^ T cell repertoire, even if their primary amino acid sequence is not closely homologous with that of F-MLV env. Furthermore, the functional avidity of env_124-138_L-reactive CD4^+^ T cells could also be affected by additional genetic determinants other than endogenous retroviruses. To address this question we generated congenic EF4.1 mice on the 129S8 background. 129S8 mice share the same MHC class II allele with B6 mice (H2-A^b^), thus allowing restriction of env_124-138_L-specific EF4.1 CD4^+^ T cells. However, they do differ substantially with respect to the composition of endogenous retroviruses and, importantly, 129S8 mice are naturally devoid of endogenous ecotropic MLVs [34], [35]. Similar frequency of env_124-138_L-reactive Vα2 CD4^+^ T cells developed in B6 and 129S8 EF4.1 mice (Figure 8A). In contrast, the frequency of env_124-138_L-reactive non-Vα2 CD4^+^ T cells was significantly higher on the 129S8 than on the B6 background (Figure 8A), and was comparable with that on the *Emv2*-deficient B6 background (Figure 5B), as was their functional avidity (Figure 8B). This finding indicated that deletion of env_124-138_L-reactive non-Vα2 CD4^+^ T cells in B6, but not in B6-*Emv2* ^−/−^ or 129S8 mice was mediated primarily by *Emv2*. Surprisingly, however, the functional avidity of env_124-138_L-reactive Vα2 CD4^+^ T cells in 129S8 mice was very much reduced in comparison with that of Vα2 CD4^+^ T cells in B6 mice (Figure 8B), and was as low as that of low-avidity non-Vα2 CD4^+^ T cells. As, a result of differences in frequency and functional avidity, the env_124-138_L-specific response of 129S8 mice was dominated by non-Vα2 CD4^+^ T cells at all peptide doses, in contrast to that of B6 mice, which was dominated by Vα2 CD4^+^ T cells at low peptide doses (Figure 8C).

To further explore the origin of high-avidity env_124-138_L-reactive Vα2 CD4^+^ T cells in B6, but not in 129S8 mice, we tested the response of a series of B6×129S8-EF4.1 F_1_ mice. In comparison with B6×129S8-EF4.1 F_1_ mice, which inherited *Emv2* from the B6 parent, B6-*Emv2* ^−/−^×129S8-EF4.1 F_1_ mice, which lacked ecotropic MLVs, had elevated frequencies of env_124-138_L-reactive non-Vα2 CD4^+^ T cells, whereas frequencies of env_124-138_L-reactive Vα2 CD4^+^ T cells were similar (Figure 8D). These results confirmed that elevated frequencies of env_124-138_L-reactive non-Vα2 CD4^+^ T cells in 129S8 mice were indeed due to lack of *Emv2*-mediated selection. Interestingly, both B6×129S8-EF4.1 and B6-*Emv2* ^−/−^×129S8-EF4.1 F_1_ mice generated env_124-138_L-reactive Vα2 CD4^+^ T cells with higher avidity than those of 129S8 mice (Figure 8E), suggesting that a genetic contribution of the B6 parent, other than *Emv2*, was necessary for the development of high-avidity env_124-138_L-reactive Vα2 CD4^+^ T cells. To assess whether this genetic contribution arose from polymorphisms in the *Trav* locus itself, we tested B6-*Tcra* ^−/−^×129S8-EF4.1 F_1_ mice, which inherited *Emv2* from the B6 parent, but could generate endogenous Vα chains only from the locus inherited from the 129S8 parent. The presence of *Emv2* in B6-*Tcra* ^−/−^×129S8-EF4.1 F_1_ mice had the predicable effect on the frequency of env_124-138_L-reactive non-Vα2 CD4^+^ T cells (Figure 8D), which displayed comparably low avidity in all three F_1_ strains tested (Figure 8E). Surprisingly, however, env_124-138_L-reactive Vα2 CD4^+^ T cells that had developed in B6-*Tcra* ^−/−^×129S8-EF4.1 F_1_ mice were also low-avidity, which was comparable with that of Vα2 CD4^+^ T cells in 129S8 mice (Figure 8E), suggesting that the ability of B6 mice to generate high-avidity env_124-138_L-reactive Vα2 CD4^+^ T cells was germline-encoded. Consequently, the env_124-138_L-specific response of B6×129S8-EF4.1 F_1_ mice, but not of isogenic mice lacking either *Emv2* or the B6-origin *Trav*, was dominated by Vα2 CD4^+^ T cells at low peptide doses (Figure 8F). The peak percentage of Vα2 CD4^+^ T cells in the env_124-138_L-reactive population was lower in B6×129S8-EF4.1 F_1_ mice than in B6 mice, as the former were expressing endogenous Vα chains from both parental *Trav* loci. Thus, the combined effect of *Emv2* on the frequency of non-Vα2 T cells and of *Trav* on the avidity of Vα2 T cells was necessary for the dominance of high-avidity Vα2 CD4^+^ T cells in the response to env_124-138_L.

## Discussion

As a result of the combinatorial process that creates TCRs, their specificity is random and has to undergo selection. Thymic positive and negative selection of developing T cells ensures that mature T cells in the periphery have a functional TCR and minimal reactivity to self proteins, respectively [36]. Negative selection is thought to decrease the frequency, avidity and cross-reactivity of the developing TCR repertoire specific to foreign epitopes that may be similar to self-derived epitopes presented in the thymus [36] and promote peptide specificity [37]. Here we used a well-characterized molecular system to show that negative selection by a defined self peptide from *Emv2* env indeed decreased the frequency in the naïve CD4^+^ T cell repertoire of clones specific to a range of foreign env epitopes, thus reducing the magnitude of the CD4^+^ T cell response to all env epitope variants. However, negative selection counter-intuitively also promoted the avidity of the CD4^+^ T cell response to F-MLV env by shifting the clonal composition of responding CD4^+^ T cells in favor of high-avidity cells.

CD4^+^ T cells play a central coordinating role in the orchestration of adaptive immunity to infection, and may also mediate direct antiviral activity. Recent studies in diverse systems have indicated an essential role for the CD4^+^ T cell response in the control of retroviral infection [15], [38]–[42]. We have previously shown that protection of wt mice against acute FV infection is proportional to the frequency of virus-specific CD4^+^ T cells [23]. Surprisingly, we found that although negative selection significantly reduced both the precursor frequency and peak expansion of F-MLV env-specific CD4^+^ T cells, it did not compromise CD4^+^ T cell-mediated antiviral activity. This finding suggested that not all virus-specific CD4^+^ T cells were equal in their ability to mediate antiviral functions. Indeed, negative selection by *Emv2* env affected CD4^+^ T cells with low avidity for F-MLV env, but not those with high avidity for the same epitope. Preservation of full antiviral activity in the *Emv2*-selected CD4^+^ T cell repertoire therefore indicated that this activity is primarily, if not exclusively, exerted by high-avidity CD4^+^ T cells.

High-avidity virus-specific CD4^+^ T cells may be superior in certain direct antiviral or indirect helper functions than low-avidity ones, but there may also be important exceptions. High-avidity CD4^+^ T cells responding to FV infection have been reported to show enhanced *ex vivo* production of IFN-γ and IL-21 cytokines and reduced expression of PD-1 inhibitory receptor [15] than low-avidity counterparts, properties that may contribute to superior antiviral activity. However, T follicular helper (Tfh) differentiation and function were previously found to be similar between high- and low-avidity virus-specific CD4^+^ T cells [15], suggesting that provision of T cell help for the production of virus-neutralizing antibodies may be more sensitive to the frequency of virus-specific CD4^+^ T cells, rather than their avidity. However, in addition to the frequency of virus-specific CD4^+^ T cells, the virus-specific antibody response is also proportional to the frequency of rare antigen-specific B cells. Thus, when availability of T cell help is abundant, the virus-specific antibody response may be limited by the frequency of antigen-specific B cells and additional T cell help would not be expected to enhance antibody production. Consistent with this idea, adoptive transfer of virus-specific EF4.1 CD4^+^ T cells into wt B6 mice did not accelerate the virus-neutralizing antibody response [23]. In addition to an effect of *Emv2* on the availability of T cell help for the FV-specific antibody response, *Emv2* could in principle also directly affect the development of virus-specific B cells [43]. Although we observed comparably low FV-specific antibody responses between B6 and B6-*Emv2* ^−/−^ mice at the peak of FV infection, our results did not exclude a potential direct effect of *Emv2* on FV-specific B cell and antibody responses at later time-points, when these responses are fully induced. Indeed, *Emv2*-encoded env shares 79% amino acid identity with F-MLV env and it is therefore possible that *Emv2* expression, especially when upregulated, might affect the FV-specific antibody response.

As previously shown, high-avidity F-MLV env_122-141_L-specific Vα2 CD4^+^ T cells are a minority subset in the naïve repertoire and only dominate the immune response to FV as a result of their preferential expansion during infection [15]. We have now found that for this ability of high-avidity F-MLV env_122-141_L-specific Vα2 CD4^+^ T cells to dominate the peak response, negative selection by *Emv2* of at least some of the competitor low-avidity F-MLV env_122-141_L-specific non-Vα2 CD4^+^ T cells is necessary. These findings indicate that even subtle thymic events can have profound effects on the induction of an effective T cell response to retroviral infection. Recently, a comprehensive theoretical study has indicated that *HLA* class I alleles that associated with control of HIV infection, such as HLA-B*5701, sample far fewer self peptides than other *HLA* alleles [5]. As a result of less stringent negative selection, a higher frequency of CD8^+^ T cells restricted by these protective alleles were predicted to recognize viral peptide epitopes and to cross-react with variants of the targeted epitopes [5].

Our results with a single self peptide provide further experimental confirmation of negative selection reducing both the precursor frequency and cross-reactivity of env-specific CD4^+^ T cells, although in this case the effect on cross-reactivity was more pronounced at the population, rather than the single-cell level. These results also suggest that from the thousands of self peptides that can mediate thymic selection of retrovirus-specific T cells, the main effects may be mediated by only a few self peptides. Moreover, self peptides with such strong influence may also be polymorphic between different individuals, which might contribute to the partial association of *HLA* polymorphisms with virus control [3], [5], [6].

In addition to polymorphisms at the *MHC/HLA* locus or of self peptides mediating thymic selection, the *Trav/TRAV* and *Trbv/TRBV* loci may also display allelic sequence variation. A polymorphism in the *TRBV9* gene has been shown to affect TCR affinity for and functional recognition of an HLA-B*3501-restricted epitope from the EBNA-1 protein of Epstein-Barr virus (EBV), leading to a public T cell response dominated by the high-affinity variant [44]. Similarly, we found that the ability of Vα2 chains to confer high avidity for env_122-141_L in EF4.1 mice seems to be germline-encoded, as only Vα2 chains encoded by the B6, but not the 129 *Trav* locus had this ability. It is tempting to speculate that amino acid residues unique to the B6-germline *Trav14*-encoded Vα2 chains participate in recognition of the strongly interacting L (or a limited set of amino acids with similar properties) at env position 128. Notably, the CD8^+^ T cell response to an HLA-B8-restricted epitope from the latent antigen EBNA 3A of EBV uses almost exclusively identical Vα and Vβ, as well as other TCR-region sequences, and comprehensive structural studies have shown that a unique amino acid residue in the germline-encoded complementarity-determining region 2 (CDR2) of the preferred Vα chain, encoded by *TRAV26-2*, is critically required for binding to a residue from the peptide epitope [45]. Despite the vast number of somatically-generated random TCRs that can arise during T cell development, these studies highlight the potential for germline-encoded residues to provide exquisite specificity and competitive advantage to the TCRs that carry them.

In addition to likely representing the best-fit for recognition of A^b^-restricted env_122-141_L, the dominance of Vα2 EF4.1 CD4^+^ T cells could also result from preferential pairing of the transgenic TCRβ chain with Vα2 chains in general. This is unlikely to be the case as the usage of Vα2 cells was not increased in either total or env_122-141_L-reactive EF4.1 CD4^+^ T cells, and indeed in the env_122-141_L-reactive preimmune repertoire clones using other Vα chains were at least 3 times more frequent than those using Vα2. However, although non-Vα2 env_122-141_L-reactive CD4^+^ T cells were still the majority in *Emv2*-expressing mice, their ability to participate in the response to FV and compete with env_122-141_L-reactive Vα2 CD4^+^ T cells was severely compromised by *Emv2*. Thus, the dominance of Vα2 CD4^+^ T cells in the response to FV infection can be seen as a combination of germline-encoded advantage in A^b^-restricted env_122-141_L recognition conferred to Vα2 CD4^+^ T cells and of *Emv2*-mediated self-tolerance of other non-Vα2 CD4^+^ T cells capable of recognizing A^b^-restricted env_122-141_L.

One important novel insight of the current study is the proof of principle that negative selection is not necessarily always impairing high-avidity T cell responses. By counter-selecting some cross-reactive CD4^+^ T cells, negatively selecting self peptides have the ability to significantly enhance the avidity for the response to at least some epitope variants. Higher precursor frequency and cross-reactivity with emerging epitope variants seem to be the best correlates for an effective cytotoxic CD8^+^ T cell response [5]. Whether higher avidity for the primary infecting epitope, rather than cross-reactivity with epitope variants better describes an effective CD4^+^ T cell response to retroviral infection needs to be further addressed.

It should be noted that differences in avidity for antigen in this system were defined functionally. Indeed, Vα2 env_122-141_L-specific primary CD4^+^ T cells or hybridomas reacted to much lower concentrations of env_122-141_L peptide stimulation *in vitro* than their non-Vα2 counterparts. Furthermore, this higher sensitivity translated to higher *in vivo* expansion and increased potential for cytokine production [15]. It is currently unclear whether differences in functional avidity between Vα2 and non-Vα2 env_122-141_L-specific CD4^+^ T cells resulted from overall higher affinity of individual TCRs of these polyclonal populations for the peptide-MHC class II complex. Although dissociation kinetics between TCRs and peptide-MHC class II tetramers are often informative with respect to the biochemical affinity of these TCRs, they may not be universally useful. For example, the available env_123-141_-A^b^ tetramer (A^b^-env) is known to bind only some env_124-138_L-specific CD4^+^ T cell clones but not others, irrespective of their functional avidity or Vα usage [14], [22]. Therefore, this reagent could not be used to access the biochemical affinity of all env_124-138_L-specific CD4^+^ T cells in the polyclonal repertoire. Furthermore, identification of antigen-specific cells using a sensitive two-dimensional binding assay has recently demonstrated that the affinity of many CD4^+^ T cells that participate in the response to two separate antigens is below detection with peptide-MHC class II tetramers [46]. Thus, peptide-MHC class II tetramers may generally only detect some but not all antigen-specific CD4^+^ T cells. In addition, such detection is conditional on expression of sufficient TCR levels. Indeed, we have found that the extensive, antigen-induced downregulation of their TCR *in vivo*, eclipses detection with the A^b^-env_123-141_ tetramer of even the env_122-141_L-reactive CD4^+^ T cells that could otherwise bind this reagent. Similar observations have been recently made with peptide-MHC class I tetramer staining of virus-specific effector CD8^+^ T cells [47], suggesting that the inability of peptide-MHC multimers to identify antigen-specific effector T cells that have downregulated their TCRs may be a general problem for T cells restricted by both classes of MHC molecules.

Negative selection ensures minimal reactivity of developing thymocytes to self proteins. However, endogenous retroviruses are a large constituent of mammalian genomes and thus represent a potentially large pool of self proteins able to mediate selection, both positive and negative. Self peptides encoded by endogenous MLVs have been shown to mediate positive selection of CD4^+^ T cells with specificity for an unrelated H2-E^k^-restricted moth cytochrome C peptide, and to enhance the response of mature CD4^+^ T cells with this specificity in the periphery [48]. We found that *Emv2* was expressed at very low levels in the thymus of B6 mice, in agreement with a previous report [49], and was undetectable by qRT-PCR in some of the mice. It should be noted, however, that the qRT-PCR method employed was specific only for the spliced *env* mRNA that is transcribed by *Emv2*. This was chosen to eliminate the possibility of detecting contaminating genomic DNA or viral genomic RNA, but may underestimate the total amount of spliced and unspliced mRNA that leads to the production of other viral proteins. Nevertheless, as demonstrated by its effect on thymic development, this low level of *Emv2* expression was clearly functional.

Endogenous retroviruses have been known for many years to cause a range of different diseases in mice, including cancer, immunodeficiency and autoimmunity, although a similar causal effect in humans has been questioned [50]. Immune reactivity to endogenous retroviruses has been amply demonstrated in mice where is has been strongly associated with the development of spontaneous autoimmune conditions [51], [52]. Interestingly, immune reactivity to endogenous retroviruses has also been frequently observed in humans during infection, inflammation, autoimmunity and cancer [50], [53]–[56]. Expression of human endogenous retroviruses, as well as CD8^+^ T cell responses against their antigens, have been documented in HIV infection [57], [58]. Furthermore, a whole-genome association study has suggested that part of the effect of the protective HLA-B*5701 allele during the asymptomatic period of HIV infection may be mediated by a linked human endogenous retrovirus at the same locus [59]. Human endogenous retroviral antigens have also been reported to serve as targets for CD8^+^ T cell-mediated rejection of cancer cells [60]. It might be evident from the studies in humans and the results of the current study that peptide epitopes encoded by endogenous retroviruses have a strong influence on T cell thymic selection and may also participate in the shaping of the peripheral T cell response. It is also clear that endogenous retroviruses do not always cause immunological tolerance, and although their activation in infected or transformed cells may provide a non-mutable target for immune attack, activation of endogenous retroviruses may also trigger inflammatory or autoimmune phenomena frequently associated with infection and cancer. Further study of endogenous retrovirus regulation during infection, autoimmunity or cancer, and of the immune responsiveness to them should shed more light into their pathogenic potential.

## Materials and Methods

### Ethics statement

All animal experiments were approved by the ethical committee of the NIMR, and conducted according to local guidelines and UK Home Office regulations under the Animals Scientific Procedures Act 1986 (ASPA).

### Mice

Inbred C57BL/6J (B6), A/J and B6.SJL-*Ptprc^a^ Pep3^b^*/BoyJ (CD45.1^+^ B6) mice were originally obtained from The Jackson Laboratory (Bar Harbor, Maine, USA) and were subsequently maintained at NIMR animal facilities. Inbred 129S8/SvEvNimrJ (129S8) mice were developed from an 129/Sv substrain, maintained at NIMR animal facilities, and were subsequently deposited at The Jackson Laboratory. The B6 TCRβ-transgenic strain EF4.1, expressing a transgenic TCRβ chain from a T cell clone specific to F-MuLV env_122-141_ presented by H2-A^b^, has been described [14]. 129S8-congenic EF4.1 mice were generated by serial backcrossing of B6-EF4.1 mice for 10 nuclear generations onto the 129S8 genetic background. B6-backcrossed Rag1-deficient (*Rag1* ^−/−^) mice [61] and T cell receptor α-deficient (*Tcra^−/−^*) mice [62] were also maintained at NIMR animal facilities. *Fv2* ^s^-congenic B6 (*Fv2^s^*) and *Rag1^−/−^* (*Fv2^s^ Rag1^−/−^*) mice have been previously described [25]. *Emv2*-deficient (*Emv2^−/−^*) B6 mice were created by introducing the *Emv2* integration site of chromosome 8 from the A/J strain, which lacks this proviral integration, by serial backcrossing for at least 12 nuclear generations onto the B6 genetic background. Lack of *Emv2* was validated by PCR for both the *D8Mit49* microsatellite marker close to the locus that detects polymorphisms in A/J (*Emv2* ^−^) and B6 (*Emv2* ^+^) strains of mice (*D8Mit49* forward 5′-TCTGTGCATGGCTGTGTATG-3′ and *D8Mit49* reverse 5′-TGGTGTGCTGCTGATGCT-3′), and also for the actual integration site using three primers, two of which were flanking the integration site (forward 5′-ACCCACTAAGTAACCCAGGCTGCCTCAGCT-3′ and reverse 5′-GACCAGAATAGAAAGACGTTCAAGTGAGCT-3′) and one located in the *Emv2* LTR (5′-ATCAGCTCGCTTCTCGCTTCTGTACCCGCG-3′) (Figure S3).

### *In vitro* T cell activation

Spleen or lymph node single-cell suspensions were prepared from EF4.1 mice and 5×10^5^ cells per well were stimulated in 96-well plates with the indicated amount of env peptide variants. The frequency of env-reactive cells in stimulated CD4^+^ T cells was defined as the frequency of cells that responded to 18-hr stimulation, before cell division or death had occurred, by upregulating CD69 expression. Correct identification of env-reactive CD4^+^ T cells by CD69 upregulation was confirmed in control experiments by co-staining for CD154 (CD40L) expression in stimulated T cells. Both antibodies were obtained from eBiosciences. For assessment of T cell activation on day 3, cells were labeled with CFSE before stimulation and responding cells were identified by CFSE dilution.

### Hybridoma cell line generation and stimulation

Single-cell suspensions were prepared from spleens and lymph nodes from *Emv2*-sufficient or -deficient EF4.1 mice and stimulated *in vitro* with 10^−7^ M or 10^−5^ M env_122-141_L peptide and 4 ng/ml recombinant human IL-2 for 4 days. CD4^+^ T cells were subsequently purified from stimulated cultures using immunomagnetic positive selection (StemCell Technologies, Vancouver, BC, Canada) and fused to TCRαβ-negative BW5147 thymoma cells to produce hybridoma cell lines. Established hybridoma cell lines were stimulated with a range of env peptide variants presented by dendritic cells. Dendritic cells were obtained from cultures of bone marrow cells isolated from B6 mice and supplemented with granulocyte macrophage colony-stimulating factor (GM-CSF). GM-CSF was obtained from culture supernatant of ×63 cells transfected with mouse *Csf2* and was used at 1∶10 dilution. Bone marrow cells were culture in these conditions for 7 days, at which point they consisted of 50–70% dendritic cells. These cells were then used to stimulate hybridoma cells at a ratio of 5×10^4^ dendritic cells to 1×10^5^ hybridoma cells, for 18 hrs, in the presence or absence of env peptide variants. Dendritic cell-hybridoma cell co-cultures were plated in flat-bottom 96-well plates in 200 µl final volume. The concentration of peptides used is indicated in individual figures and figure legends. In additional experiments peritoneal macrophages were also used as antigen-presenting cells with results comparable to the use of dendritic cells. Macrophages were isolated from B6 mice following plating of the peritoneal cavity exudate cells for 1 hr and washing off the non-adherent fraction. Env-specific responses were assessed by measuring the amount of IL-2 secreted in co-culture supernatants using an AlamarBlue (Invitrogen, Carlsbad, CA, USA)-based CTLL-2 assay.

### *Tra* gene usage

*Trav* and *Traj* usage by T cell hybridomas was probed by staining with an anti-Vα2 (clone B20.1) or anti-Vα3.2 (clone RR3-16) monoclonal antibodies, and by reverse transcription (RT)-PCR amplification and sequencing of expressed *Trav* genes, using previously described primers [63]. *Trav* and *Traj* segment identification and alignment, and confirmation of productive rearrangements were performed on the International Immunogenetics Information System website (http://www.imgt.org).

### Viruses and infections

The FV used in this study was a retroviral complex of a replication-competent B-tropic F-MuLV and a replication-defective polycythemia-inducing spleen focus-forming virus (SFFVp). Stocks were propagated *in vivo* and prepared as 10% w/v homogenate from the spleen of 12-day infected BALB/c mice. Mice received an inoculum of ∼1,000 spleen focus-forming units of FV. All viral stocks were free of Sendai virus, Murine hepatitis virus, Parvoviruses 1 and 2, Reovirus 3, Theiler's murine encephalomyelitis virus, Murine rotavirus, Ectromelia virus, Murine cytomegalovirus, K virus, Polyomavirus, Hantaan virus, Murine norovirus, Lymphocytic choriomeningitis virus, Murine adenoviruses FL and K87, and Lactate dehydrogenase-elevating virus. Virus inocula were injected via the tail vein in 0.1 ml of phosphate-buffered saline. FV-infected cells were detected by flow cytometry using surface staining for the glycosylated product of the viral *gag* gene (glyco-Gag), using the matrix (MA)-specific monoclonal antibody 34 (mouse IgG2b), followed by an anti-mouse IgG2b-FITC secondary reagent (BD, San Jose, CA, USA). For the assessment of anemia, mice were bled by a small incision of the tail vein and blood was collected into heparinized capillary tubes. Complete blood counts were measured on a VetScan HMII hematology analyzer (Abaxis, CA, USA), following the manufacturer's instructions. RBC counts of uninfected mice were ∼9.95×10^6^ per mm^3^ of blood. FV-induced splenomegaly in infected mice was expressed as spleen index, which is the ratio of the weight of the spleen (in mg) to the weight of the rest of the body (in g).

### FV-neutralizing and F-MLV-infected cell-binding antibody assays

Serum titers of FV-neutralizing antibodies were measured as previously described [25]. The dilution of serum which resulted in 75% neutralization was taken as the neutralizing titer. Serum titers of F-MLV-infected cell-binding antibodies were determined by flow cytometry following primary staining of F-MLV-infected *Mus dunni* cells with serial dilutions of serum samples and secondary staining with fluorescently labeled anti-mouse IgG1 (clone A85-1), anti-mouse IgG2a/c (clone R19-15), anti-mouse IgG2b (clone R12-3) or anti-mouse IgM (clone R6-60.2) antibodies (BD). B6 mice express the IgG2c isotype, which may not be efficiently detected by anti-IgG2a reagents [64]. Although the R19-15 monoclonal antibody has higher affinity for IgG2a, it can be effectively used for detection of IgG2c. This was confirmed by staining of F-MLV-infected *Mus dunni* cells that were first incubated with serum from FV-infected mice, with the anti-IgG1 or anti-IgG2a/c or anti-IgG2b reagents separately (Figure S8A). The three reagents used separately resulted in comparable staining intensity, which allowed us to use all three IgG subclass-specific antibodies in combination. For IgG titers, F-MLV-infected *Mus dunni* cells were first incubated with serum samples and then with anti-IgG1, anti-IgG2a/c and anti-IgG2b antibodies mixed together. Serum samples were 2-fold serially diluted, starting from an initial dilution of 1∶50. The last positive serum dilution resulting in staining intensity at least twice the background level was taken as the binding titer (Figure S8B).

### T cell purification and adoptive transfer

Single-cell suspensions were prepared from the spleens and lymph nodes of donor CD45.2^+^ EF4.1 mice by mechanical disruption. Spleen suspensions were treated with ammonium chloride for erythrocyte lysis. CD4^+^ T cells were enriched using immunomagnetic positive selection (StemCell Technologies) according to the manufacturer's instructions. Purity of the isolated CD4^+^ T-cell population was routinely higher than 92%. A total of approximately 1×10^6^ EF4.1 CD4^+^ T cells were injected in B6-congenic CD45.1^+^CD45.2^+^ recipients via the tail vein in 0.1 ml of air-buffered Iscove's Modified Dulbecco's Media. When adoptive transfer of CD4^+^ T cells was combined with FV infection, purified CD4^+^ T cells and virus stocks were injected separately into recipient mice within a 24 hour-period.

### Flow cytometry

Spleen-cell suspensions were stained with directly-conjugated antibodies to surface markers, obtained from eBiosciences (San Diego, CA, USA), CALTAG/Invitrogen, BD Biosciences (San Jose, CA, USA) or BioLegend (San Diego, CA, USA). Donor-type env-specific CD4^+^ T cells were identified as CD44^hi^CD45.2^+^CD45.1^−^CD4^+^ cells. Four- and 8-color cytometry were performed on FACSCalibur (BD Biosciences) and CyAn (Dako, Fort Collins, CO) flow cytometers, respectively, and analyzed with FlowJo v8.7 (Tree Star Inc., Ashland, OR, USA) or Summit v4.3 (Dako) analysis software, respectively.

### *Emv2* expression by quantitative reverse transcription (qRT)-PCR

Total RNA was extracted from whole organs using TRI-reagent (Sigma-Aldrich, St. Louis, US) according to the manufacturer's instructions, precipitated with isopropanol and washed in 75% ethanol before being dissolved in water. DNase digestion and cleanup was performed with the RNeasy Mini Kit (Qiagen, Hilden, Germany) and cDNA produced with the high capacity reverse transcription kit (Applied Biosystems, Carlsbad, US) with an added RNase inhibitor (Promega Biosciences, Madison, US). A final clean-up was performed with the QIAquick PCR purification kit (Qiagen). Level of expression of *Emv2* RNA was determined by qRT-PCR using DNA Master SYBR Green I kit (Roche, Mannheim, Germany) and the ABI Prism 7000 or 7900HT Detection System (TaqMan, Applied Biosystems, Foster City, CA). The following primers were used for the amplification of target transcripts: *Hprt*: forward 5′-TTGTATACCTAATCATTATGCCGAG-3′ and reverse 5′- CATCTCGAGCAAGTCTTTCA-3′; *Emv2*: forward 5′-CCAGGGACCACCGACCCACCGT-3′ and reverse 5′-TAGTCGGTCCCGGTAGGCCTCG-3′. *Emv2*-specific primers amplified only the spliced form of *env* mRNA, thus eliminating the possibility of residual genomic DNA or RNA contamination contributing to *Emv2* signal. The housekeeping gene *Hprt* was used to normalize the Critical Threshold (C_T_) values for *Emv2*. Analysis was conducted with the ΔC_T_ method [65] and *Emv2* expression corresponding to an *Emv2* C_T_ value of 40 (the total number of amplification cycles used) was set at 1 arbitrary unit. A theoretical detection limit of 2 arbitrary units was also used, which represents the detectable *Emv2* signal in the penultimate cycle of amplification.

### Statistical analysis

Statistical comparisons were made using SigmaPlot 12.0 (Systat Software Inc., Germany). Parametric comparisons of normally-distributed values that satisfied the variance criteria were made by unpaired Student's *t*-tests. Linear percentages of FV-infected cells, spleen indices and nAb titers, which did not pass the variance test, were compared with non-parametric two-tailed Mann-Whitney Rank Sum or Wilcoxon Signed Rank tests.

### Accession numbers

- *Cd4* cluster of differentiation 4 antigen [*Mus musculus*]; Gene ID: 12504; Protein ID: NP_038516.1
- *Rag1* recombination activating gene 1 [*Mus musculus*]; Gene ID: 19373; Protein ID: NP_033045.2
- *Fv2* Friend virus susceptibility 2 [*Mus musculus*]; Gene ID: 19882; Protein ID: NP_033100.1
- *Tcra* T cell receptor alpha chain [*Mus musculus*]; Gene ID: 21473
- *Tcrb* T cell receptor beta chain [*Mus musculus*]; Gene ID: 21577
- *Emv2* endogenous ecotropic MuLV 2 [*Mus musculus*]; Gene ID: 111372
- *env* envelope protein [*Friend murine leukemia virus*]; Gene ID: 1491875; Protein ID: NP_040334.1

## Supporting Information

Figure S1 **Effect of N-terminal epitope length on TCR recognition by primary and hybridoma EF4.1 envL-specific CD4^+^ T cells.** (A) Frequency of CD69^+^ cells in Vα2 or non-Vα2 CD4^+^ T cells (expressed as percentage of the maximal response elicited by the env_122-141_L peptide), following 18-hr *in vitro* stimulation of spleen cell suspensions from EF4.1 mice with the indicated range of N-terminal truncated envL peptides. (B) IL-2 production in the supernatant of hybridoma cells lines established from Vα2 or non-Vα2 env-specific EF4.1 CD4^+^ T clones following 24-hr *in vitro* stimulation with the indicated range of N-terminal truncated envL peptides. Data are pooled from 3 separate experiments.
(PDF)

Figure S2 **Sequence and TCR SB14-31 contact residues of F-MLV- and** ***Emv2*** **-encoded env_123-140_.** (A) Amino acid sequence, in single-letter code, of env_123-140_ encoded by either F-MLV or *Emv2*. Differences in sequence are indicated by red color. (B) Important contact residues for the SB14-31 TCR (indicated in red) and for H2-A^b^ (indicated in blue) in F-MLV-encoded env_123-140_L. Numbers underneath amino acid residues correspond to amino acid positions in env.
(PDF)

Figure S3 **Chromosomal location of** ***Emv2*** **in B6 mice and screening of** ***Emv2*** **^−/−^ B6 mice.** *Emv2* is integrated near the telomere of Chromosome 8 of B6 mice in reverse orientation relative to the forward strand, between the *Tubb3* and *Def8* genes (Search for *Mela* on http://www.ncbi.nlm.nih.gov/mapview), and it is absent from A/J mice. Lack of *Emv2* on *Emv2* ^−/−^ congenic B6 mice is shown by PCR for the actual integration site (*red arrows*) or for the polymorphic D8Mit49 microsatellite marker that is further telomeric with respect to *Emv2* (*not shown on map*).
(PDF)

Figure S4 **Effect of** ***Emv2*** **on the endogenous CD4^+^ T cell, CD8^+^ T cell and antibody responses.** B6 and B6-*Emv2* ^−/−^ mice were infected with FV and their adaptive responses were measured 7 days later. T cell responses were measured in cells isolated from the spleens and antibody responses from the sera of these mice. (A) Percentage of CD44^hi^CD43^+^ cells in total CD8^+^ T cells. (B) Percentage of Vα3.2^+^Vβ5.2^+^ cells in either CD44^hi^ (*left*) or CD44^lo^ (*right*) CD8^+^ T cells. The dashes horizontal line represents the same frequency in uninfected control mice. The dashed horizontal lines in (A) and (B) represent the depicted frequencies in uninfected control mice. (C) Serum titers of F-MLV-infected cell-binding IgG (*left*) and IgM (*right*). Dashed lines represent the limit of detection. (D) Percentage of A^b^-env_123-141_ tetramer^+^ cells in total CD4^+^ T cells. Horizontal short lines denote the median frequencies and the dashed line denotes the median frequency of A^b^-hCLIP (control) tetramer^+^ cells in the same populations. (E) Percentage of Vα2 cells in A^b^-env_123-141_ tetramer^+^ CD4^+^ T cells from the same mice. The dashed horizontal line represents the frequency of Vα2 cells in total CD4^+^ T cells from the same mice. In (A) to (E) each symbol represents an individual mouse.
(PDF)

Figure S5 **Gating strategy for the identification of env-specific donor CD4^+^ T cells.** CD45.2^+^ (*Ptprc* ^2/2^) EF4.1 CD4^+^ T cells (10^6^) were adoptively transferred into wild-type *Ptprc* ^1/2^ B6 recipients that were infected with FV the same day. Host cells were identified as CD45.1 CD45.2 double-positive whereas donor cells were CD45.2 single-positive.
(PDF)

Figure S6 **Effect of** ***Emv2*** **on the frequency and composition of env_124-138_L-specific CD4^+^ thymocytes.** Thymocytes from *Emv2* ^+/+^ or *Emv2* ^−/−^ EF4.1 mice were stimulated for 18 hrs *in vitro* with the indicated amount of env_124-13_L peptide presented by bone marrow-derived dendritic cells and responding cells were identified by upregulation of CD69 expression. Frequency of responding (CD69^+^) cells in gated CD4^+^ thymocytes (*left*) and frequency of Vα2 cells in env_124-13_L-specific cells (*right*) is shown, with *p*<0.006 and *p*<0.002, respectively, for 10^−6^ M peptide concentration. Results are the means ± SEM (*n* = 8–10).
(PDF)

Figure S7 **Depth of env epitope recognition by** ***Emv2*** **-selected and -nonselected T cell hybridomas.** Vα2 or non-Va2 (Vα3) env_124-138_L-reactive T cell hybridomas were established from *Emv2* ^+/+^ or *Emv2* ^−/−^ EF4.1 mice and tested for reactivity against a library of env_126-138_ peptide epitopes (at 5×10^−6^ M concentration), in which positions 128, 129 and 133 were individually replaced by all natural amino acids. The response of each clone was measured by secretion of IL-2 and is expressed as a percentage of the maximal response obtained with the most potent variant. Results are the means of triplicate cultures.
(PDF)

Figure S8 **Determining titers of F-MLV-infected cell-binding antibodies.** (A) *Mus dunni* cells, chronically infected with F-MLV, were stained with an 1∶50 dilution of pooled serum samples from wt B6 mice that were infected with FV 35 days earlier (serum sample) or with a dilution of the anti-gp70 of F-MLV monoclonal antibody 48 (mAb 48; IgG2a) that gives similar *in vitro* FV neutralization as 1∶50 dilution of the serum sample. Cells stained with the serum sample were subsequently stained with anti-IgG1, anti-IgG2a/c or anti-IgG2c antibodies separately or with all three antibodies mixed together, and cells stained with mAb 48 were stained with anti-IgG2a/c. Black-filled histograms show staining with both primary and secondary antibodies, and gray-shaded histograms show staining with the secondary antibody only. Note comparable staining intensity between all three IgG subclass-specific antibodies used separately as secondary reagents in combination with the serum sample and also in comparison with the mAb 48. (B) Example of titer determination. Experimental serum samples were serially diluted 2-fold (starting from 1∶50) and used for staining F-MLV-infected *Mus dunni* cells. The median fluorescent intensity (MFI) of co-staining with all three IgG subclass-specific antibodies at the same time is plotted against the serum dilution. The horizontal dashed line represents the MFI of unstained cells. Data were fitted to a sigmoidal curve. The red lines connect the MFI that is twice the background level and the serum dilution that results in that MFI. The inverse of the serum dilution that results in an MFI at least twice the background level was taken as the titer. This was preferred over titer determination based on half the maximal response, as in none of the samples were an obvious maximum (plateau of the curve) reached.
(PDF)

Table S1 **Va3.2 expression (determined by FACS),** ***Trav*** **and** ***Traj*** **segment usage, and amino acid residues at the VJ junction of env_124-138_L-reactive hybridoma T cell lines generated from** ***Emv2*** **^+/+^ EF4.1 mice.**
(PDF)

Table S2 **Va3.2 expression (determined by FACS),** ***Trav*** **and** ***Traj*** **segment usage, and amino acid residues at the VJ junction of env_124-138_L-reactive hybridoma T cell lines generated from** ***Emv2*** **^−/−^ EF4.1 mice.**
(PDF)
