# Supplementary material for: Negative Selection by an Endogenous Retrovirus Promotes a Higher-Avidity CD4+ T Cell Response to Retroviral Infection
Source: PLoS Pathog. 2012 May 10;8(5):e1002709. doi: 10.1371/journal.ppat.1002709 (PMC3349761; doi:10.1371/journal.ppat.1002709)
Supplement: Figure S1 — Effect of N-terminal epitope length on TCR recognition by primary and hybridoma EF4.1 envL-specific CD4+ T cells. (A) Frequency of CD69+ cells in Vα2 or non-Vα2 CD4+ T cells (expressed as percentage of the maximal response elicited by the env122-141L peptide), following 18-hr in vitro stimulation of spleen cell suspensions from EF4.1 mice with the indicated range of N-terminal truncated envL peptides. (B) IL-2 production in the supernatant of hybridoma cells lines established from Vα2 or non-Vα2 env-specific EF4.1 CD4+ T clones following 24-hr in vitro stimulation with the indicated range of N-terminal truncated envL peptides. Data are pooled from 3 separate experiments. (PDF) [file ppat.1002709.s001.pdf]

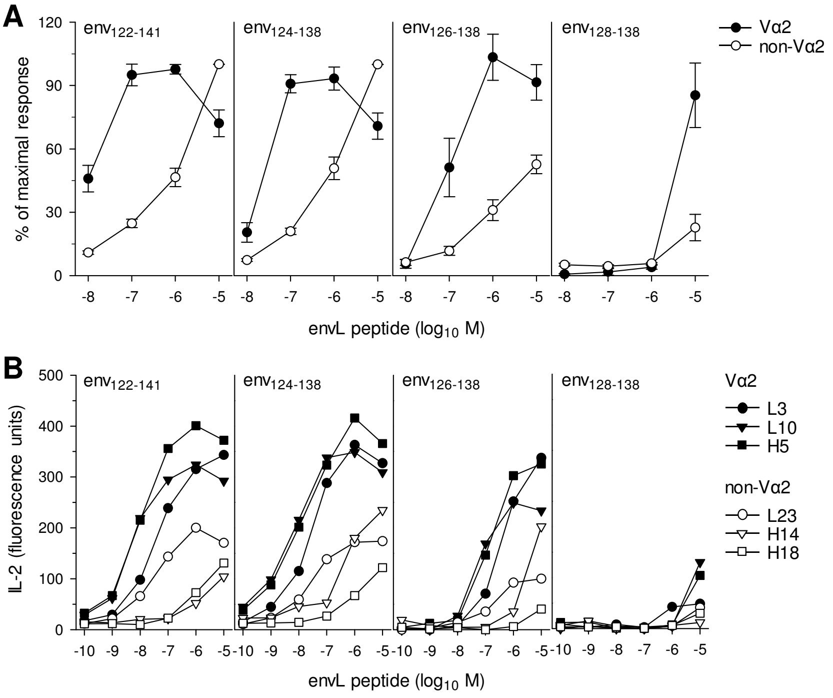

Figure S1. Effect of N-terminal epitope length on TCR recognition by primary and hybridoma EF4.1 envL-specific CD4$^+$ T cells.

(A) Frequency of CD69$^+$ cells in Vα2 or non-Vα2 CD4$^+$ T cells (expressed as percentage of the maximal response elicited by the env$_{122\text{-}141}$L peptide), following 18-hr *in vitro* stimulation of spleen cell suspensions from EF4.1 mice with the indicated range of N-terminal truncated envL peptides. (B) IL-2 production in the supernatant of hybridoma cells lines established from Vα2 or non-Vα2 env-specific EF4.1 CD4$^+$ T clones following 24-hr *in vitro* stimulation with the indicated range of N-terminal truncated envL peptides. Data are pooled from 3 separate experiments.
