# Supplementary material for: Negative Selection by an Endogenous Retrovirus Promotes a Higher-Avidity CD4+ T Cell Response to Retroviral Infection
Source: PLoS Pathog. 2012 May 10;8(5):e1002709. doi: 10.1371/journal.ppat.1002709 (PMC3349761; doi:10.1371/journal.ppat.1002709)
Supplement: Figure S2 — Sequence and TCR SB14-31 contact residues of F-MLV- and Emv2 -encoded env123-140. (A) Amino acid sequence, in single-letter code, of env123-140 encoded by either F-MLV or Emv2. Differences in sequence are indicated by red color. (B) Important contact residues for the SB14-31 TCR (indicated in red) and for H2-Ab (indicated in blue) in F-MLV-encoded env123-140L. Numbers underneath amino acid residues correspond to amino acid positions in env. (PDF) [file ppat.1002709.s002.pdf]

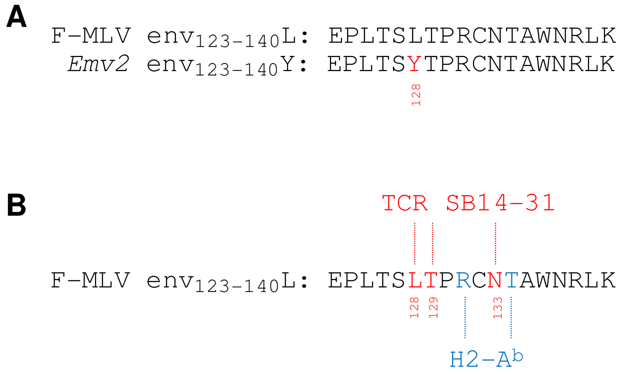

Figure S2. Sequence and TCR SB14-31 contact residues of F-MLV- and *Emv2*-encoded env$_{123\text{-}140}$.
(A) Amino acid sequence, in single-letter code, of env$_{123\text{-}140}$ encoded by either F-MLV or *Emv2*. Differences in sequence are indicated by red color. (B) Important contact residues for the SB14-31 TCR (indicated in red) and for H2-A^{b} (indicated in blue) in F-MLV-encoded env$_{123\text{-}140}$L. Numbers underneath amino acid residues correspond to amino acid positions in env.
