# Supplementary material for: Negative Selection by an Endogenous Retrovirus Promotes a Higher-Avidity CD4+ T Cell Response to Retroviral Infection
Source: PLoS Pathog. 2012 May 10;8(5):e1002709. doi: 10.1371/journal.ppat.1002709 (PMC3349761; doi:10.1371/journal.ppat.1002709)
Supplement: Figure S3 — Chromosomal location of Emv2 in B6 mice and screening of Emv2 −/− B6 mice. Emv2 is integrated near the telomere of Chromosome 8 of B6 mice in reverse orientation relative to the forward strand, between the Tubb3 and Def8 genes (Search for Mela on http://www.ncbi.nlm.nih.gov/mapview), and it is absent from A/J mice. Lack of Emv2 on Emv2 −/− congenic B6 mice is shown by PCR for the actual integration site (red arrows) or for the polymorphic D8Mit49 microsatellite marker that is further telomeric with respect to Emv2 (not shown on map). (PDF) [file ppat.1002709.s003.pdf]

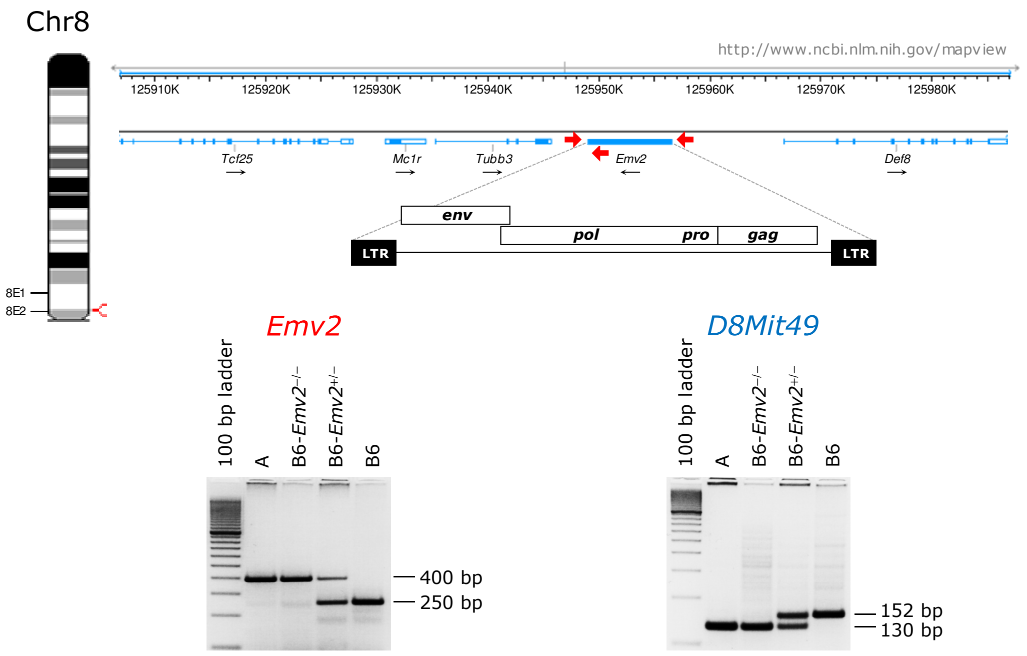


**Figure S3. Chromosomal location of *Emv2* in B6 mice and screening of *Emv2*$^{-/-}$ B6 mice.**

*Emv2* is integrated near the telomere of Chromosome 8 of B6 mice in reverse orientation relative to the forward strand, between the *Tubb3* and *Def8* genes (Search for *Mela* on http://www.ncbi.nlm.nih.gov/mapview), and it is absent from A/J mice. Lack of *Emv2* on *Emv2*$^{-/-}$ congenic B6 mice is shown by PCR for the actual integration site (*red arrows*) or for the polymorphic D8Mit49 microsatellite marker that is further telomeric with respect to *Emv2* (*not shown on map*).
