# Supplementary material for: Negative Selection by an Endogenous Retrovirus Promotes a Higher-Avidity CD4+ T Cell Response to Retroviral Infection
Source: PLoS Pathog. 2012 May 10;8(5):e1002709. doi: 10.1371/journal.ppat.1002709 (PMC3349761; doi:10.1371/journal.ppat.1002709)
Supplement: Figure S4 — Effect of Emv2 on the endogenous CD4+ T cell, CD8+ T cell and antibody responses. B6 and B6-Emv2 −/− mice were infected with FV and their adaptive responses were measured 7 days later. T cell responses were measured in cells isolated from the spleens and antibody responses from the sera of these mice. (A) Percentage of CD44hiCD43+ cells in total CD8+ T cells. (B) Percentage of Vα3.2+Vβ5.2+ cells in either CD44hi (left) or CD44lo (right) CD8+ T cells. The dashes horizontal line represents the same frequency in uninfected control mice. The dashed horizontal lines in (A) and (B) represent the depicted frequencies in uninfected control mice. (C) Serum titers of F-MLV-infected cell-binding IgG (left) and IgM (right). Dashed lines represent the limit of detection. (D) Percentage of Ab-env123-141 tetramer+ cells in total CD4+ T cells. Horizontal short lines denote the median frequencies and the dashed line denotes the median frequency of Ab-hCLIP (control) tetramer+ cells in the same populations. (E) Percentage of Vα2 cells in Ab-env123-141 tetramer+ CD4+ T cells from the same mice. The dashed horizontal line represents the frequency of Vα2 cells in total CD4+ T cells from the same mice. In (A) to (E) each symbol represents an individual mouse. (PDF) [file ppat.1002709.s004.pdf]

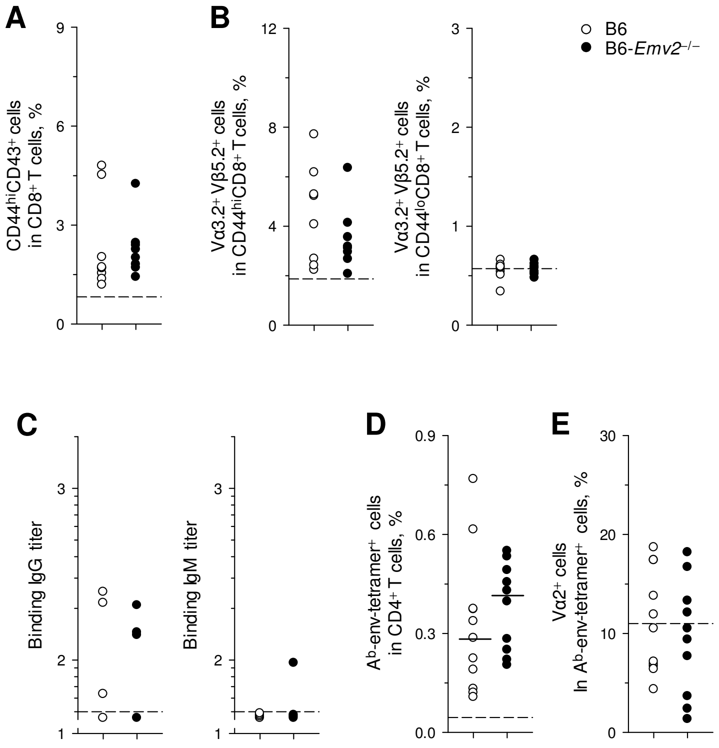

Figure S4. Effect of *Emv2* on the endogenous $CD4^+$ T cell, $CD8^+$ T cell and antibody responses.

B6 and B6-$Emv2^{-/-}$ mice were infected with FV and their adaptive responses were measured 7 days later. T cell responses were measured in cells isolated from the spleens and antibody responses from the sera of these mice. (A) Percentage of $CD44^{hi}CD43^+$ cells in total $CD8^+$ T cells. (B) Percentage of $V\alpha3.2^+V\beta5.2^+$ cells in either $CD44^{hi}$ (*left*) or $CD44^{lo}$ (*right*) $CD8^+$ T cells. The dashes horizontal line represents the same frequency in uninfected control mice. The dashed horizontal lines in (A) and (B) represent the depicted frequencies in uninfected control mice. (C) Serum titers of F-MLV-infected cell-binding IgG (*left*) and IgM (*right*). Dashed lines represent the limit of detection. (D) Percentage of A^b-$env_{123-141}$ tetramer$^+$ cells in total $CD4^+$ T cells. Horizontal short lines denote the median frequencies and the dashed line denotes the median frequency of A^b-hCLIP (control) tetramer$^+$ cells in the same populations. (E) Percentage of $V\alpha2$ cells in A^b-$env_{123-141}$ tetramer$^+$ $CD4^+$ T cells from the same mice. The dashed horizontal line represents the frequency of $V\alpha2$ cells in total $CD4^+$ T cells from the same mice. In (A) to (E) each symbol represents an individual mouse.
