# Supplementary material for: Negative Selection by an Endogenous Retrovirus Promotes a Higher-Avidity CD4+ T Cell Response to Retroviral Infection
Source: PLoS Pathog. 2012 May 10;8(5):e1002709. doi: 10.1371/journal.ppat.1002709 (PMC3349761; doi:10.1371/journal.ppat.1002709)
Supplement: Figure S5 — Gating strategy for the identification of env-specific donor CD4+ T cells. CD45.2+ (Ptprc 2/2) EF4.1 CD4+ T cells (106) were adoptively transferred into wild-type Ptprc 1/2 B6 recipients that were infected with FV the same day. Host cells were identified as CD45.1 CD45.2 double-positive whereas donor cells were CD45.2 single-positive. (PDF) [file ppat.1002709.s005.pdf]

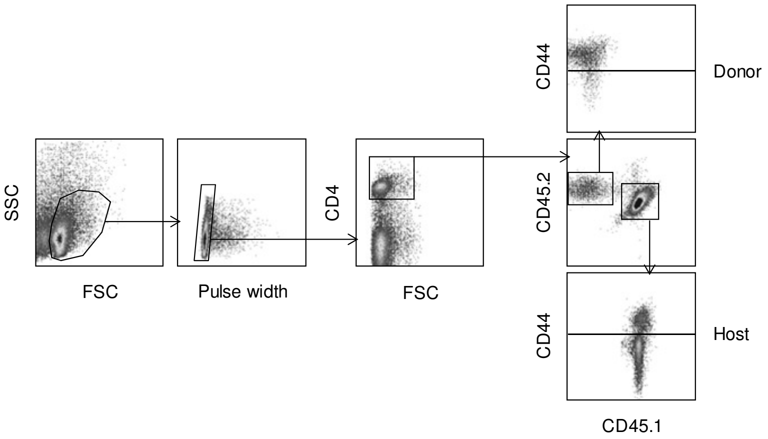

Figure S5. Gating strategy for the identification of env-specific donor CD4⁺ T cells.

CD45.2$^+$ (*Ptprc*$^{2/2}$) EF4.1 CD4$^+$ T cells (10^6) were adoptively transferred into wild-type *Ptprc*$^{1/2}$ B6 recipients that were infected with FV the same day. Host cells were identified as CD45.1 CD45.2 double-positive whereas donor cells were CD45.2 single-positive.
