# Supplementary material for: Negative Selection by an Endogenous Retrovirus Promotes a Higher-Avidity CD4+ T Cell Response to Retroviral Infection
Source: PLoS Pathog. 2012 May 10;8(5):e1002709. doi: 10.1371/journal.ppat.1002709 (PMC3349761; doi:10.1371/journal.ppat.1002709)
Supplement: Figure S6 — Effect of Emv2 on the frequency and composition of env124-138L-specific CD4+ thymocytes. Thymocytes from Emv2 +/+ or Emv2 −/− EF4.1 mice were stimulated for 18 hrs in vitro with the indicated amount of env124-13L peptide presented by bone marrow-derived dendritic cells and responding cells were identified by upregulation of CD69 expression. Frequency of responding (CD69+) cells in gated CD4+ thymocytes (left) and frequency of Vα2 cells in env124-13L-specific cells (right) is shown, with p<0.006 and p<0.002, respectively, for 10−6 M peptide concentration. Results are the means ± SEM (n = 8–10). (PDF) [file ppat.1002709.s006.pdf]

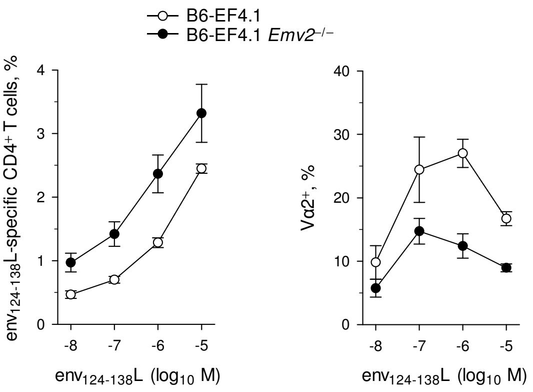

Figure S6. Effect of *Emv2* on the frequency and composition of env$_{124\text{-}138}$L-specific CD4$^+$ thymocytes.

Thymocytes from *Emv2*$^{+/+}$ or *Emv2*$^{-/-}$ EF4.1 mice were stimulated for 18 hrs *in vitro* with the indicated amount of env$_{124\text{-}13}$L peptide presented by bone marrow-derived dendritic cells and responding cells were identified by upregulation of CD69 expression. Frequency of responding (CD69$^+$) cells in gated CD4$^+$ thymocytes (*left*) and frequency of Vα2 cells in env$_{124\text{-}13}$L-specific cells (*right*) is shown, with p < 0.006 and p < 0.002, respectively, for 10^{-6} M peptide concentration. Results are the means ± SEM (n = 8-10).
