# Supplementary material for: Negative Selection by an Endogenous Retrovirus Promotes a Higher-Avidity CD4+ T Cell Response to Retroviral Infection
Source: PLoS Pathog. 2012 May 10;8(5):e1002709. doi: 10.1371/journal.ppat.1002709 (PMC3349761; doi:10.1371/journal.ppat.1002709)
Supplement: Figure S7 — Depth of env epitope recognition by Emv2 -selected and -nonselected T cell hybridomas. Vα2 or non-Va2 (Vα3) env124-138L-reactive T cell hybridomas were established from Emv2 +/+ or Emv2 −/− EF4.1 mice and tested for reactivity against a library of env126-138 peptide epitopes (at 5×10^−6^ M concentration), in which positions 128, 129 and 133 were individually replaced by all natural amino acids. The response of each clone was measured by secretion of IL-2 and is expressed as a percentage of the maximal response obtained with the most potent variant. Results are the means of triplicate cultures. (PDF) [file ppat.1002709.s007.pdf]

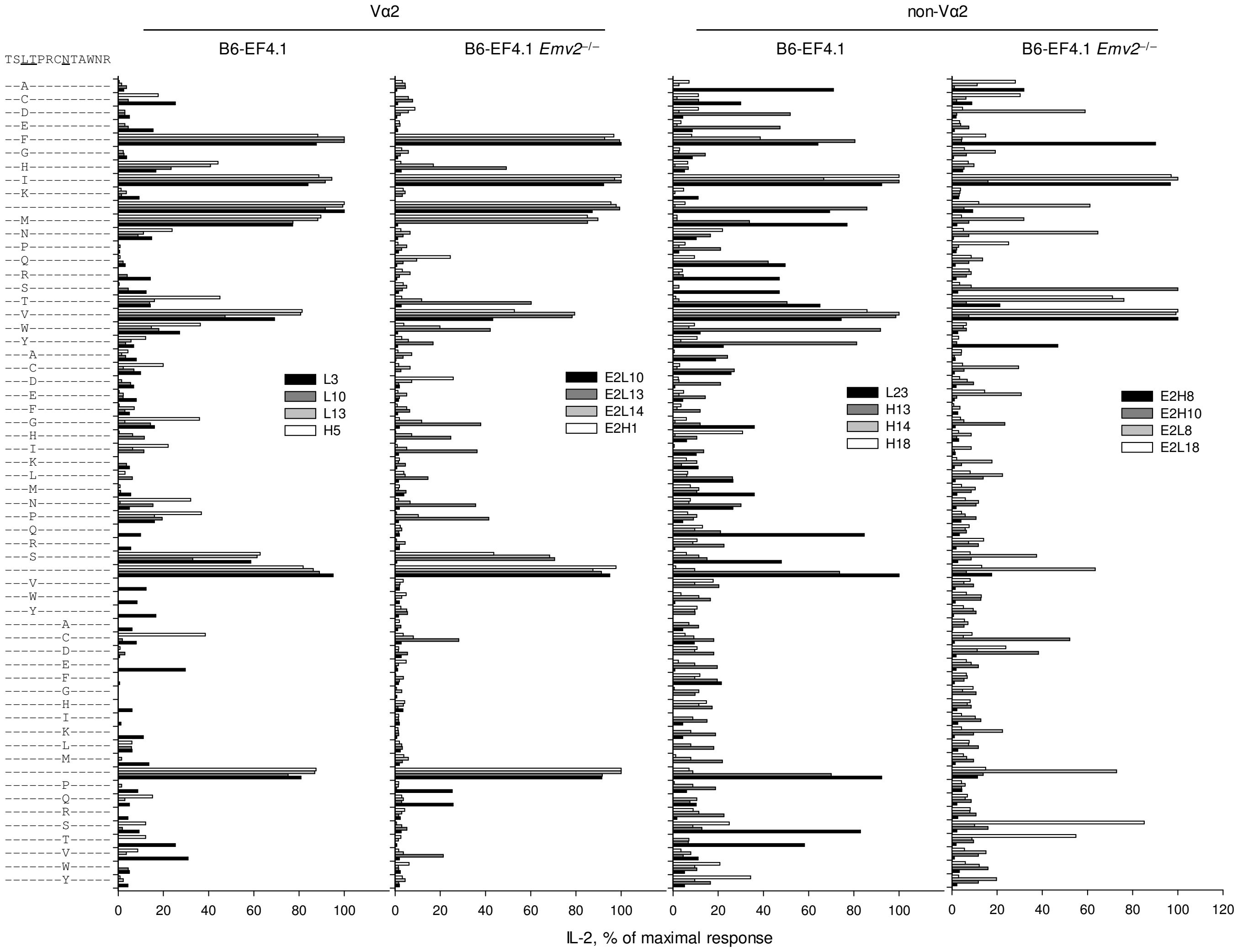

Figure S7. Depth of env epitope recognition by *Emv2*-selected and -nonselected T cell hybridomas. Vα2 or non-Va2 (Vα3) $env_{124\text{-}138}$L-reactive T cell hybridomas were established from $Emv2^{+/+}$ or $Emv2^{-/-}$ EF4.1 mice and tested for reactivity against a library of $env_{126\text{-}138}$ peptide epitopes (at 5×10^{-6} M concentration), in which positions 128, 129 and 133 were individually replaced by all natural amino acids. The response of each clone was measured by secretion of IL-2 and is expressed as a percentage of the maximal response obtained with the most potent variant. Results are the means of triplicate cultures.
