# Supplementary material for: Negative Selection by an Endogenous Retrovirus Promotes a Higher-Avidity CD4+ T Cell Response to Retroviral Infection
Source: PLoS Pathog. 2012 May 10;8(5):e1002709. doi: 10.1371/journal.ppat.1002709 (PMC3349761; doi:10.1371/journal.ppat.1002709)
Supplement: Figure S8 — Determining titers of F-MLV-infected cell-binding antibodies. (A) Mus dunni cells, chronically infected with F-MLV, were stained with an 1∶50 dilution of pooled serum samples from wt B6 mice that were infected with FV 35 days earlier (serum sample) or with a dilution of the anti-gp70 of F-MLV monoclonal antibody 48 (mAb 48; IgG2a) that gives similar in vitro FV neutralization as 1∶50 dilution of the serum sample. Cells stained with the serum sample were subsequently stained with anti-IgG1, anti-IgG2a/c or anti-IgG2c antibodies separately or with all three antibodies mixed together, and cells stained with mAb 48 were stained with anti-IgG2a/c. Black-filled histograms show staining with both primary and secondary antibodies, and gray-shaded histograms show staining with the secondary antibody only. Note comparable staining intensity between all three IgG subclass-specific antibodies used separately as secondary reagents in combination with the serum sample and also in comparison with the mAb 48. (B) Example of titer determination. Experimental serum samples were serially diluted 2-fold (starting from 1∶50) and used for staining F-MLV-infected Mus dunni cells. The median fluorescent intensity (MFI) of co-staining with all three IgG subclass-specific antibodies at the same time is plotted against the serum dilution. The horizontal dashed line represents the MFI of unstained cells. Data were fitted to a sigmoidal curve. The red lines connect the MFI that is twice the background level and the serum dilution that results in that MFI. The inverse of the serum dilution that results in an MFI at least twice the background level was taken as the titer. This was preferred over titer determination based on half the maximal response, as in none of the samples were an obvious maximum (plateau of the curve) reached. (PDF) [file ppat.1002709.s008.pdf]

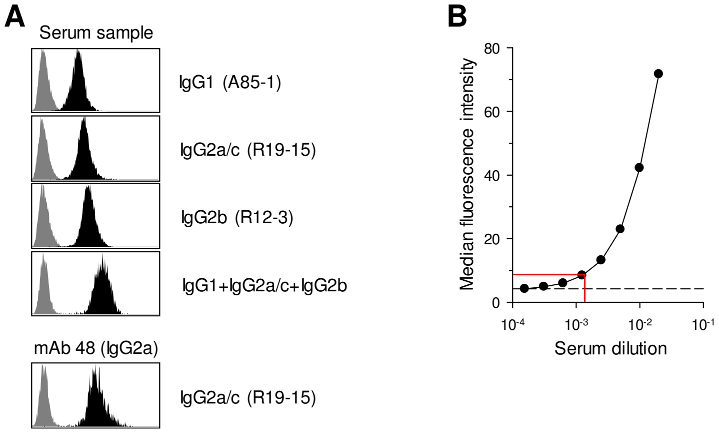

Figure S8. Determining titers of F-MLV-infected cell-binding antibodies.

(A) *Mus dunni* cells, chronically infected with F-MLV, were stained with an 1:50 dilution of pooled serum samples from wt B6 mice that were infected with FV 35 days earlier (serum sample) or with a dilution of the anti-gp70 of F-MLV monoclonal antibody 48 (mAb 48; IgG2a) that gives similar *in vitro* FV neutralization as 1:50 dilution of the serum sample. Cells stained with the serum sample were subsequently stained with anti-IgG1, anti-IgG2a/c or anti-IgG2c antibodies separately or with all three antibodies mixed together, and cells stained with mAb 48 were stained with anti-IgG2a/c. Black-filled histograms show staining with both primary and secondary antibodies, and gray-shaded histograms show staining with the secondary antibody only. Note comparable staining intensity between all three IgG subclass-specific antibodies used separately as secondary reagents in combination with the serum sample and also in comparison with the mAb 48. (B) Example of titer determination. Experimental serum samples were serially diluted 2-fold (starting from 1:50) and used for staining F-MLV-infected *Mus dunni* cells. The median fluorescent intensity (MFI) of co-staining with all three IgG subclass-specific antibodies at the same time is plotted against the serum dilution. The horizontal dashed line represents the MFI of unstained cells. Data were fitted to a sigmoidal curve. The red lines connect the MFI that is twice the background level and the serum dilution that results in that MFI. The inverse of the serum dilution that results in an MFI at least twice the background level was taken as the titer. This was preferred over titer determination based on half the maximal response, as in none of the samples were an obvious maximum (plateau of the curve) reached.
