# Supplementary material for: Negative Selection by an Endogenous Retrovirus Promotes a Higher-Avidity CD4+ T Cell Response to Retroviral Infection
Source: PLoS Pathog. 2012 May 10;8(5):e1002709. doi: 10.1371/journal.ppat.1002709 (PMC3349761; doi:10.1371/journal.ppat.1002709)
Supplement: Table S1 — Va3.2 expression (determined by FACS), Trav and Traj segment usage, and amino acid residues at the VJ junction of env124-138L-reactive hybridoma T cell lines generated from Emv2 +/+ EF4.1 mice. (PDF) [file ppat.1002709.s009.pdf]

Table S1. Va3.2 expression (determined by FACS), *Trav* and *Traj* segment usage, and amino acid residues at the VJ junction of $env_{124\text{-}138}$L-reactive hybridoma T cell lines generated from *Emv2*$^{+/+}$ EF4.1 mice.

Clone name	Va3.2 expression	TRAV	TRAJ	AA Junction
H13	+	9D-4*04	31*01	CAVEGGNNRIFF
H14	-	9N-4*01	17*01	CALSNSAGNKLTF
H18	+	9N-2*01	31*01	CVLSRNSNNRIFF
L23	-	9N-4*01	27*01	CALDNTNTGKLTF
