# Supplementary material for: Negative Selection by an Endogenous Retrovirus Promotes a Higher-Avidity CD4+ T Cell Response to Retroviral Infection
Source: PLoS Pathog. 2012 May 10;8(5):e1002709. doi: 10.1371/journal.ppat.1002709 (PMC3349761; doi:10.1371/journal.ppat.1002709)
Supplement: Table S2 — Va3.2 expression (determined by FACS), Trav and Traj segment usage, and amino acid residues at the VJ junction of env124-138L-reactive hybridoma T cell lines generated from Emv2 −/− EF4.1 mice. (PDF) [file ppat.1002709.s010.pdf]

Table S2. Va3.2 expression (determined by FACS), *Trav* and *Traj* segment usage, and amino acid residues at the VJ junction of $env_{124\text{-}138}$L-reactive hybridoma T cell lines generated from *Emv2*$^{-/-}$ EF4.1 mice.

Clone name	Va3.2 expression	TRAV	TRAJ	AA Junction
E2H8	-	9N-4*01	17*01	CALSNSAGNKLTF
E2H10	-	9N-4*01	21*01	CALSGPNYNVLYF
E2L8	-	9N-2*01	16*01	CVLSGSSSGQKLVF
E2L18	+	9N-3*01	58*01	CAVRQGTGSKLSF
